# Initial Findings from a Novel School-Based Program, EMPATHY, Which May Help Reduce Depression and Suicidality in Youth

**DOI:** 10.1371/journal.pone.0125527

**Published:** 2015-05-14

**Authors:** Peter H. Silverstone, Marni Bercov, Victoria Y. M. Suen, Andrea Allen, Ivor Cribben, Jodi Goodrick, Stu Henry, Catherine Pryce, Pieter Langstraat, Katherine Rittenbach, Samprita Chakraborty, Rutger C. Engels, Christopher McCabe

**Affiliations:** 1 Department of Psychiatry, University of Alberta, Edmonton, Canada; 2 Strategic Clinical Network for Addiction and Mental Health, Alberta Health Services, Edmonton, Alberta, Canada; 3 Department of Finance and Statistical Analysis, University of Alberta, Edmonton, Canada; 4 Red Deer Public Schools, Red Deer, Alberta, Canada; 5 Department of Economics, Faculty of Art, University of Alberta, Edmonton, Canada; 6 Trimbos-Institute, P.O. Box 725, 3500 AS Utrecht, The Netherlands; 7 Department of Emergency Medicine and Public Health, Edmonton, Canada; United (Osaka U, Kanazawa U, Hamamatsu U Sch Med, Chiba U and Fukui U) Graduate School of Child Developmen, JAPAN

## Abstract

We describe initial pilot findings from a novel school-based approach to reduce youth depression and suicidality, the Empowering a Multimodal Pathway Towards Healthy Youth (EMPATHY) program. Here we present the findings from the pilot cohort of 3,244 youth aged 11–18 (Grades 6-12). They were screened for depression, suicidality, anxiety, use of drugs, alcohol, or tobacco (DAT), quality-of-life, and self-esteem. Additionally, all students in Grades 7 and 8 (mean ages 12.3 and 13.3 respectively) also received an 8-session cognitive-behavioural therapy (CBT) based program designed to increase resiliency to depression. Following screening there were rapid interventions for the 125 students (3.9%) who were identified as being actively suicidal, as well as for another 378 students (11.7%) who were felt to be at higher-risk of self-harm based on a combination of scores from all the scales. The intervention consisted of an interview with the student and their family followed by offering a guided internet-based CBT program. Results from the 2,790 students who completed scales at both baseline and 12-week follow-up showed significant decreases in depression and suicidality. Importantly, there was a marked decrease in the number of students who were actively suicidal (from n=125 at baseline to n=30 at 12-weeks). Of the 503 students offered the CBT program 163 (32%) took part, and this group had significantly lower depression scores compared to those who didn’t take part. There were no improvements in self-esteem, quality-of-life, or the number of students using DAT. Only 60 students (2% of total screened) required external referral during the 24-weeks following study initiation. These results suggest that a multimodal school-based program may provide an effective and pragmatic approach to help reduce youth depression and suicidality. Further research is required to determine longer-term efficacy, reproducibility, and key program elements.

**Trial Registration:**

ClinicalTrials.gov NCT02169960

## Introduction

Evidence from a variety of sources suggests that depressive disorders occur in approximately 10% of youth [1], [2], that the frequency of several mental disorders may be increasing in youth [3], and that many disorders in adulthood are first evident during youth [4]. Accompanying the high rates of depression and other mental health disorders, suicide rates are also high in youth [4–6]. Indeed, a recent large U.S. survey reported that over 7% of High-School students had attempted suicide in the previous 12 months, of which one-third required hospital treatment [7], while a large European study reported that 4% of youth have attempted suicide [8]. One way of addressing this may be to utilize school-based prevention programs [9], although more research is needed in order to draw definitive conclusions regarding the best approach to achieve this [10–14]. Among suggestions for the options to address youth suicidality, it has been suggested that a comprehensive multimodal approach may be most effective [15]. Such multimodal approaches may involve a wide variety of interventions such as increasing mental health information to students [15], screening [9], and resiliency programs based on cognitive-behavioural therapy principles, although there is a relative dearth of intervention studies [16].

It should be noted that one problem in research in this area is that scales may not accurately predict those who go on to carry out acts of self-harm [17], although a recent study suggests that two questions regarding suicidal intent contained within the 9-item patient health questionnaire (PHQ-9) may be able to do this [18]. Furthermore, in youth, the relationship between rates of self-harm and suicide rates is complex. While it is likely that less than 5% of youth who carry out self-harm will subsequently have a completed suicide [19], it is clear that those at greatest risk of completing suicide are individuals who have either previously carried out self-harming behaviours and/or who have depression [20], [21]. Therefore, one method to reduce youth suicides may be to reduce depression rates in this group.

Many of the school-based programs which have shown benefit are based upon cognitive behavioural therapy (CBT) principles. One such approach is the Penn Resiliency Program [22–25], which has been studied for over 20 years. More recently an updated version of this program, called “OVK” (so-called because of its Dutch name “Op Volle Kracht” which is translated as On Full Power), has been created in collaboration with the original developers of the Penn Resiliency Program [26]. The full OVK program is delivered during regular school lessons, and consists of 16 sessions. The first 8 lessons are CBT focused and the second 8 lessons are on aspects of social and educational learning. Several studies have found beneficial outcomes on depression rates with OVK in both the short-term and longer-term [27], [28], but positive results have not been found in all studies [29].

Several groups have suggested that the most appropriate place for interventions to reduce depression rates in youth is by intervention in schools, either for all individuals (“universal” programs) or for specific “high-risk” populations [30–34]. Universal prevention may only produce small overall changes [35], although a Cochrane review of over 50 studies concluded that “universal depression prevention programs may prevent the onset of depressive disorders compared with no intervention” [36]. The other major approach to address youth depression rates is to carry out more “targeted” approaches for those students identified as “high-risk”. This is because the large number of individuals who do not need interventions mean that many students need to be screened to identify each case [37]. Multiple different approaches have examined potential risk factors that can identify potential high-risk youth, with most utilizing existing depression scales to screen youth followed by a CBT-based program to treat depression [38–45]. However, it is likely that an additional 3–7% of youth have “sub-threshold” depression (i.e. depressive symptoms that do not meet diagnostic criteria) [46], [47], and many of these have active suicidal thoughts [8]. Thus, one of the issues with a “high-risk” approach is that if a high score on a depression screening measure is used as the only way to determine those at risk, it is likely to miss many youth who have active suicidal thoughts. Combining both a universal and a high-risk approach may potentially have the greatest impact [5], [48], although studies to date have not found such benefits [49], [50]. It is possible that other factors, such as family relationship support, may be important in the outcome of these interventions [51].

To help both prevent and treat depression in youth there is an increasing focus on technology approaches [52], [53], both a universal basis [54], and to treat youth who are depressed [55–58]. The results are generally positive [53], although it is unclear how they compare to in-person delivery of CBT [59]. Overall, one of the most consistent findings from these technology-based studies is that a “guided” approach, meaning that there is a trained individual acting as a “guide” for the user at each stage of these programs, consistently improves both uptake rates and outcomes compared to individuals just using such programs on their own [58], [60], [61]. Consistent with this are findings that simply disseminating a self-directed internet-based intervention to a school population is not effective, even if significant steps are taken to reduce barriers to use [54].

Findings to date are that there exists no single program that has demonstrated consistent, and longer-term, effectiveness on reducing suicidality and depression in youth, although the need for this remains clear [5]. The present publication describes the first results from the piloting of a novel school-based approach to reduce depression and suicidality in youth. This program is called Empowering a Multimodal Pathway Towards Healthy Youth (EMPATHY). It utilizes a multimodal approach including a universal CBT program for some, screening of all students to identify a potentially higher-risk group, rapid intervention, and a guided internet-based CBT approach for those identified as depressed or at high-risk. Our primary hypothesis was that this approach would reduce both depression and suicidality, and our secondary hypotheses were that this program would also reduce anxiety and use of drugs, alcohol, and tobacco (DAT) while increasing self-esteem and quality-of-life.

## Materials and Methods

### Ethics statement

The detailed TREND checklist detailing how the study addressed methodological issues is contained within the supporting information (S1 TREND Checklist). All aspects of this program were approved by the Health Research Ethics Committee of the University of Alberta on September 23, 2013, who approved all informed consent forms and all other aspects of the study. Subsequent amendments to the original protocol and consent letters were approved in January of 2014, with some additional minor changes to the informed consent letter subsequently being approved in February 2014, prior to the first student starting. The full original protocol submitted to the ethics committee, as well as changes approved in January 2014, is contained within the supporting information (S1 Protocol). All parents received written information that all school students would be offered screening as a standard part of their health classes, with information about this, including information sessions. However, specific written consent for this was not required (as it wasn’t within the school district for any other school-wide activity), although parents were able to have their children not be included in screening, and student participation was voluntary. For any individual student who was to be offered additional intervention specific written informed consent was required from both parents/guardians and the student. The first student was screened in February 2014 and the follow-up screening was completed by the end of June 2014.

This study is registered with ClinicalTrials.gov Identifier: NCT02169960. Although an application for this was completed at the time of the ethics approval, due to an unfortunate administrative oversight, the actual submission to the registration database did not occur until July 2014. This omission was corrected as soon as it was recognized. The authors confirm that all ongoing and related trials for this intervention are now registered.

In terms of study design, from a scientific viewpoint, the most rigorous findings would have come from a design in which a group of students were randomized to be screened, but not to have any interventions, compared to those who were screened and did have an intervention. Indeed, such an approach has been carried out in many studies looking at depression rates in students. This could have been done at the class level or school level. However, given that we were specifically screening for youth who were actively suicidal, having a group that was screened and found to be suicidal but then not offering an intervention would clearly have been an unethical approach. For this reason we determined that using each student as their own controls was an appropriate study design at this stage of the research process, and this was approved by the Health Research Ethics Committee.

### Study location and timing

We engaged with Red Deer Public Schools in May of 2013 and a widespread multi-sectoral community partnership approach was implemented involving education services, primary health care, specialist mental health care, social services, as well as others involved with youth (such as the police services). Following extensive consultation with large numbers of interested parties, researchers, and community groups, implementation started in February 2014 with follow-up data being collected by June 2014. The present study reports on these initial findings, which is the first pilot of this comprehensive program. Longer-term follow-up studies are planned, and modifications of the program are anticipated based on the present study experiences and findings. It should be emphasized that using all schools in a region (and not selecting specific ones) is a unique approach that we are not aware has been used in previous studies.

### Novel multimodal approach

Whenever possible existing screening, measurement, and intervention tools were used. To reduce costs, and improve potential future ease of uptake, only such tools that were useable at no cost were considered, and they also had to be able to be used electronically on a tablet. Additionally, all staff hired in the schools to implement the program had to have some experience working with youth, and many had an undergraduate degree. However it is important to note that they were specifically not highly trained individuals (such as psychologists or teachers), as it was felt that it would not be feasible for widespread expansion if such highly trained (and expensive) staff were required. These individuals were termed “Resiliency Coaches”, and each was attached to a specific school, becoming integrated with the staff at that school. It was made clear to all study staff and school staff that the Resiliency Coaches were not therapists or counsellors, and would not act in those roles. As noted in previous studies, obtaining “buy-in” from schools and students as well as other community members can be difficult, but is important for the success in any such school-based programs. For this reason, among others, multiple approaches were undertaken to try and increase the chances of success for the EMPATHY program, with such approaches maximizing technology whenever possible (Table 1).

**Table 1:** Some of the novel multimodal approaches utilized in EMPATHY program.

The use of electronic tablets with a specifically developed software “app” that was given within the classroom setting on a single occasion for rapid and consistent data collection during screening. This required all screening scales to be short, free, and able to be programed within this “app”.
Ultra-rapid feedback being given to schools regarding students considered at higher risk of suicide. Feedback to the school occurred within 1–2 hours, as an output from this “app”.
All students who had significant suicidal thoughts had a 1- hour interview either the same day or the following working day (for this reason little screening was carried out on Fridays), and their family was contacted immediately afterwards.
Identification of other high-risk individuals by use of measurement of multiple factors that are important in mental health to create a single summary score (the combined Resiliency scale score). Additional interventions were offered based upon this score.
The use of established internet-based CBT programs for the 10% of those identified by this process as being considered at greatest risk. These programs were administered in a “guided” manner by the Resiliency Coaches.
The use of a well-established universal resiliency CBT program (OVK), targeted initially at only 2 Grades (for budgetary and other logistical reasons). These were Grades 7 and 8 (ages 12–13) based upon previous positive results in this age group.
A 5-day integrated CBT training program was provided for the 5 Resiliency Coaches hired for this program, which included the study rationale, OVK, and the CBT internet-based programs.
Dedicated training on diagnosis and treatment approaches for community physicians and mental health staff working in primary care, including specific information on CBT and other treatment approaches for youth.
Awareness of the program in the community through communication with students, parents/guardians, and the use of various types of media including print and television.
Careful tracking of all referrals to both primary care and specialist mental health care during the program, as well as ensuring that (if required) more staff could be made available to help with any additional needs.
The ability to link outcomes to individual school attendance and achievement.
A city-wide approach involving all public schools that catered solely to Grades 6–12.

#### Screening

**Fig 1:**
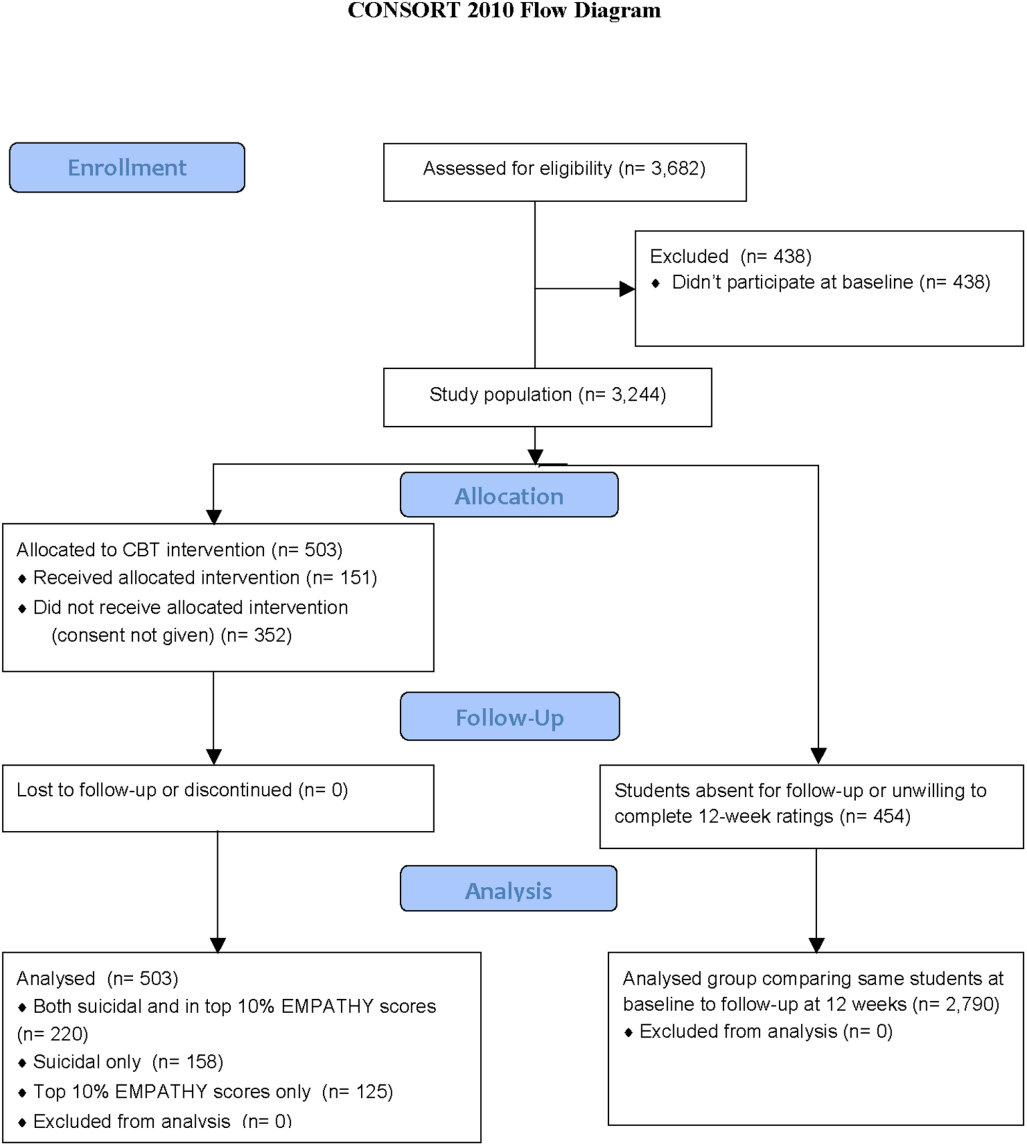
Consort flow chart demonstrating allocation of students. The figure shows how many students entered the study and (on the left-hand side) how many were allocated to the CBT treatment as well as (on the right-hand side) how many completed both baseline and 12-week follow-up ratings and formed the study population.

The allocation of students involved in this study is shown in Fig 1, including those offered interventions after screening. All screening was carried out on dedicated electronic tablets during a 45-minute period within a standard classroom session. Students logged on using only their student IDs. Electronic data collection complied with all privacy and security requirements. The individual scales were presented to students in a randomized order, and no data was stored on the tablets as they were directly linked to the school intranet. The data was stored in a dedicated and secure database, but was immediately available to the study staff to identify those at risk.

#### Screening tools

All of the screening tools met the criteria for being free, short, and easily adapted to the “app” used on the electronic tablet. The exact questions asked, and the scores associated with each, are shown in Table 2. Note that there are several minor variations from some of the published scales, and these are described below, as we were not using these scales to diagnose students, or to indicate thresholds of abuse, but to indicate areas of concern experienced by each individual. This means that the scores from each of the components cannot always be directly compared to previous studies. It is also important to note that we did not “pilot” these changes or validate them in any way. Therefore, we recognize that this approach means that where we have changed the original scales they cannot be directly compared to scores from other studies using the original unmodified scales. Each of the scales is described below.

**Table 2:** List of questions asked.

Source of question ^c^	Question Number	Stem Questions (where appropriate)	Individual Questions	Scoring Range for each question
**List of questions asked to determine depression score and suicide risk**				
HQ-9	1	Over the past 2 weeks, how often have you been bothered by:	Little interest or pleasure in doing things	0 to 3
PHQ-9	2	Over the past 2 weeks, how often have you been bothered by:	Feeling down, depressed, or hopeless	0 to 3
PHQ-9	3	Over the past 2 weeks, how often have you been bothered by:	Trouble falling or staying asleep, or sleeping too much	0 to 3
PHQ-9	4	Over the past 2 weeks, how often have you been bothered by:	Feeling tired or having little energy	0 to 3
PHQ-9	5	Over the past 2 weeks, how often have you been bothered by:	Poor appetite or over eating	0 to 3
PHQ-9	6	Over the past 2 weeks, how often have you been bothered by:	Feeling bad about yourself-or that you are a failure or have let yourself or your family down	0 to 3
PHQ-9	7	Over the past 2 weeks, how often have you been bothered by:	Trouble concentrating on things, such as reading or watching TV	0 to 3
PHQ-9	8	Over the past 2 weeks, how often have you been bothered by:	Moving or speaking so slowly that other people could have noticed. Or the opposite-being so fidgety or restless that you have been moving around a lot more than usual	0 to 3
PHQ-9 ^b^	9	Over the past 2 weeks, how often have you been bothered by:	Thoughts of hurting yourself	0 to 3
PHQ-9 ^b^	10	Over the past 2 weeks, how often have you been bothered by:	***Thoughts that you would be better off dead*** ^c^	0 to 3
PHQ-9	11	If you checked off "any problems", how difficult have these problems made it for you to do your work, take care of things at home, or get along with other people?		0 to 3
PHQ-9	12	Only if scored 1, 2, or 3 on question 9 does this question get asked	***Has there been a time in the past month when you have had serious thoughts about ending your life*?** ^**c**^	Yes or No
	13	Only if scored 1, 2, or 3 on question 9 was this question asked	***Have you ever*, *in your WHOLE LIFE*, *tried to kill yourself or made a suicide attempt*?** ^c^	Yes or No
			**Unmodified maximum possible raw score = **	**33**
**List of questions asked to determine anxiety score** ^**d**^				
HAD scale	1		I feel tense or wound up	0 to 3
HAD scale	2		I get a sort of frightened feeling as if something bad is about to happen	0 to 3
HAD scale	3		Worrying thoughts go through my mind	0 to 3
HAD scale	4		I can sit at ease and feel relaxed	0 to 3
HAD scale	5		I get a sort of frightened feeling like butterflies in the stomach	0 to 3
HAD scale	6		I feel restless and have to be on the move	0 to 3
HAD scale	7		I get sudden feelings of panic	0 to 3
			**Unmodified maximum possible raw score = **	**21**
**List of questions asked to determine drug, alcohol, and tobacco (DAT) score** ^**e**^				
CRAFFT	1		During the past 12 months, did you drink any alcohol (more than a few sips)?	0 or 1
CRAFFT	2		During the past 12 months, did you smoke any marijuana or hashish?	0 or 1
CRAFFT	3		During the past 12 months, did you use anything else to get high?	0 or 1
CRAFFT	4		During the past 12 months, have you ever ridden in a CAR driven by someone (including yourself) who was "high" or had been using alcohol or drugs?	0 or 1
CRAFFT	5		During the past 12 months, do you ever use alcohol or drugs to RELAX, feel better about yourself, or fit in?	0 or 1
CRAFFT	6		During the past 12 months, do you ever use alcohol or drugs while you are by yourself, or ALONE?	0 or 1
CRAFFT	7		During the past 12 months, do you every FORGET things you did while using alcohol or drugs?	0 or 1
CRAFFT	8		During the past 12 months, do your FAMILY or FRIENDS ever tell you that you should cut down on your drinking or drug use?	0 or 1
CRAFFT	9		During the past 12 months, have you ever gotten into TROUBLE while you were using alcohol or drugs?	0 or 1
	10		During the past 12 months, did you smoke tobacco products?	0 or 1
	11		During the past 12 months, did you use smokeless tobacco products?	0 or 1
			**Unmodified maximum possible raw score = **	**11**
**List of questions asked to determine self-esteem score**				
Rosenberg	1		On the whole, I am satisfied with myself	0–3
Rosenberg	2		At times I think I am no good at all	0–3 reversed
Rosenberg	3		I feel that I have a number of good qualities	0–3
Rosenberg	4		I am able to do things as well as most other people	0–3
Rosenberg	5		I feel I do not have much to be proud of	0–3 reversed
Rosenberg	6		I certainly feel useless at times	0–3 reversed
Rosenberg	7		I feel that I'm a person of worth, at least on an equal plane with others	0–3
Rosenberg	8		I wish I could have more respect for myself	0–3 reversed
Rosenberg	9		All in all, I am inclined to feel that I am a failure	0–3 reversed
Rosenberg	10		I take a positive attitude toward myself	0–3
			**Unmodified maximum possible raw score = **	**30** ^**f**^
KIDSCREEN	1	Thinking about the last week:	Have you physically felt fit and well?	0–4
KIDSCREEN	2	Thinking about the last week:	Have you felt full of energy?	0–4
KIDSCREEN	3	Thinking about the last week:	Have you felt sad?	0–4
KIDSCREEN	4	Thinking about the last week:	Have you felt lonely?	0–4
KIDSCREEN	5	Thinking about the last week:	Have you had enough time for yourself?	0–4
KIDSCREEN	6	Thinking about the last week:	Have you been able to do the things that you want to do in your free time?	0–4
KIDSCREEN	7	Thinking about the last week:	Have your parent(s) treated you fairly?	0–4
KIDSCREEN	8	Thinking about the last week:	Have you had fun with your friends?	0–4
KIDSCREEN	9	Thinking about the last week:	Have you got on well at school?	0–4
KIDSCREEN	10	Thinking about the last week:	Have you been able to pay attention?	0–4
KIDSCREEN	11	In general, how would you say your health is?		0–4
			**Unmodified maximum possible raw score = **	**44** ^**f**^

^a^ The original source of most of the questions used was the 9-item patient health questionnaire (PHQ-9) [62].

^b^ While we asked these two questions separately, they are a single question in the original PHQ-9.

^c^ These three questions were used to determine suicide risk.

^d^ The original source of the questions used was the 7-items regarding anxiety contained within the Hospital Anxiety and Depression Scale [66].

^e^ The original source of the questions was the CRAAFT questionnaire which is well validated for use in youth [77], [78]. It is named the CRAFFT scale because of the 6 questions relating to specific risks involved with drug and alcohol abuse (in our study they were questions 4–9, with the key words involved in the acronym shown in capital letters).

^f^ For these scales a higher score is better (for both self-esteem and quality-of-life) in contrast to the other scales.

For depression screening, we utilized questions from the 9-item patient health questionnaire (PHQ-9) [62], adapted for adolescents (PHQ-A) which has been well validated in youth [63–65]. As part of this original questionnaire, one question asks about frequency of thoughts in the past month about wanting to be dead. If the answer is positive to this question, then an additional question is asked about active suicidal ideation. These two questions have previously been shown to be predictive of subsequent suicidal behaviour [18]. To further focus on the risk of suicide, we also separated the question that asked about self-harm into two questions (Table 2). Furthermore, since it is well recognized that the most accurate predictor of actual suicide is a history of a previous suicide attempt [20], [21], we added an additional question that asked about a history of this (Table 2). In the present study, therefore, there were a maximum of 13 questions about depression, although the two additional questions were not asked of all the population and, therefore, they were not counted in the depression score (but were used to identify suicide risk—see below).

We utilized the questions from the anxiety section of the Hospital Anxiety and Depression Scale [66] to screen for anxiety symptoms (Table 2). It has previously been used to measure anxiety in youth in several studies [67–69].

To measure self-esteem we utilized the questions contained in the Rosenberg Self-Esteem scale [70], which has been widely used in youth [71–73] (Table 2).

To measure quality-of-life we utilized questions from the KIDSCREEN-10 (Table 2). This is recent measure of well-being and quality of life developed internationally for children and adolescents aged 8 to 18 years old [74]. It is a short version of the KIDSCREEN-52 and KIDSCREEN-27 instruments and has demonstrated validity with this population [75]. In the KIDSCREEN-10 items are measured usually on a 5-point Likert scale and summed into a score that is transformed into standard values based upon data from an international survey sample and range from -16.06 to 57.77 [76]. Because we used a composite score in this study, only the raw data was used, namely the scores from all 11 questions (Table 2).

In the program we also measured the use of drugs, alcohol, and tobacco (DAT) (Table 2). To do this we utilized the questions contained in the CRAAFT questionnaire, which is well validated for use in youth [77], [78]. However, we also used answers to all 9 questions, as this indicated use of DAT, and not simply the answers to the 6 subsequent questions which indicate risk of abuse (Table 2). Additionally, we wanted to be aware of tobacco use in this population, including smokeless tobacco use, and for this reason we asked two additional questions to those of the CRAAFT. These were “during the past 12 months did you smoke tobacco products?” and “during the past 12 months did you use smokeless tobacco products?”. For this item, therefore, there were 11 separate questions each of which was scored equally (Table 2).

#### Identification of the “actively suicidal” group

**Fig 2:**
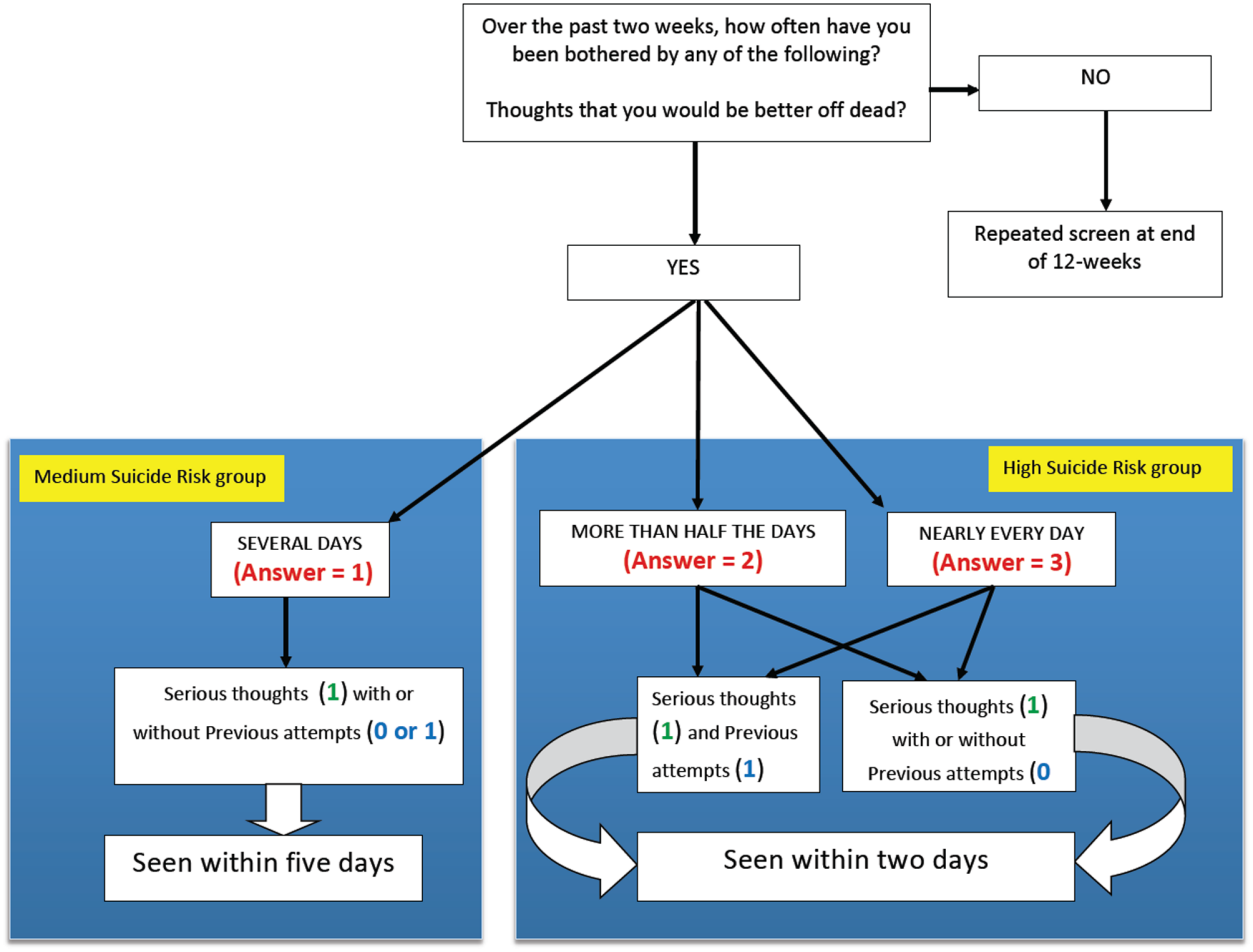
Algorithm for determining which students were in the “actively suicidal” group. The figure shows how scores on the three questions regarding suicide risk determined if the student was in the high suicide risk group or the medium suicide risk group. Together, these students were considered the “actively suicidal" group, and were interviewed individually within 2 days for the high suicide risk group or within 5 days for the medium suicide risk group.

**Fig 3:**
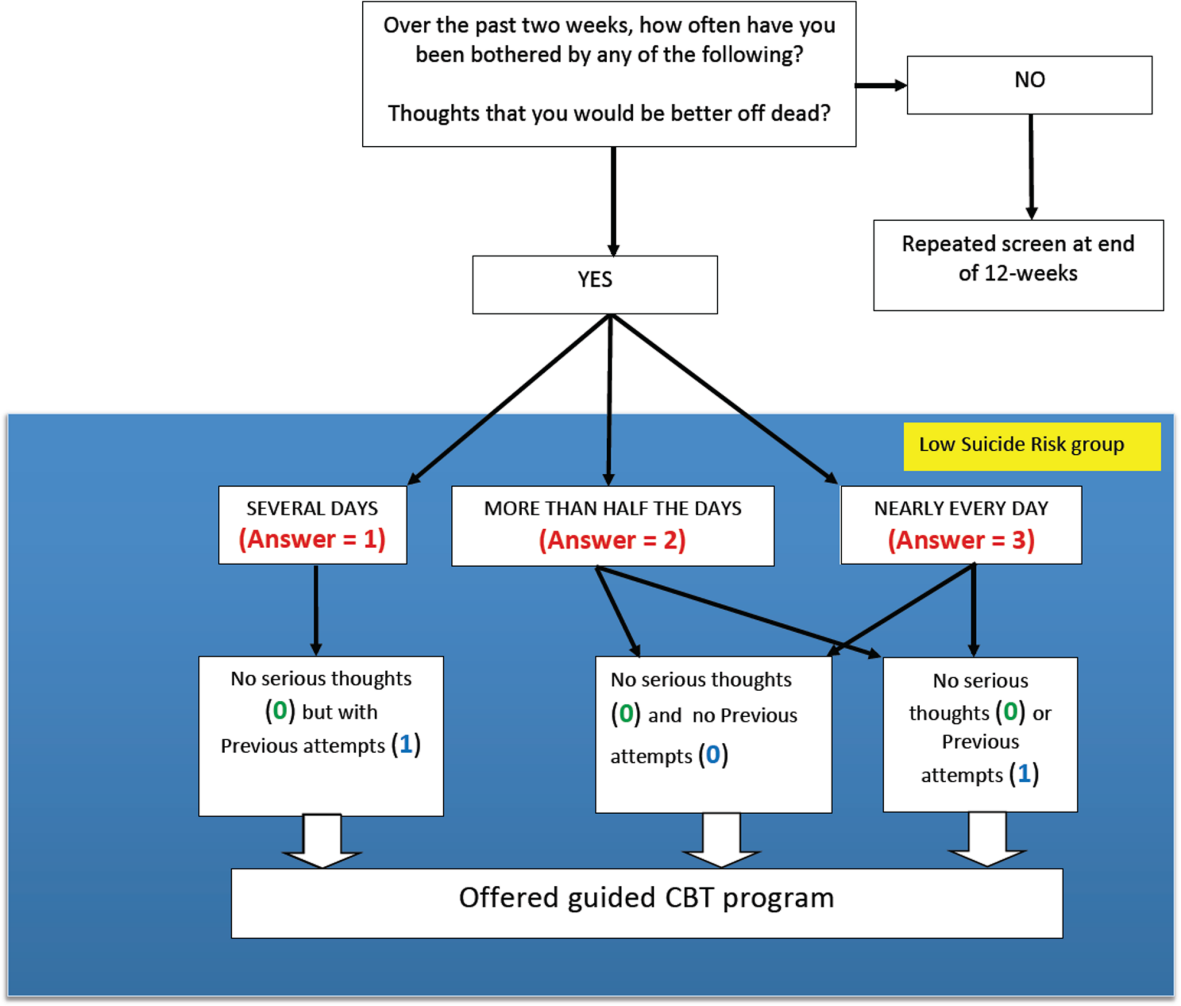
Algorithm for determining which students were in the “low suicide risk” group. The figure shows how scores on the three questions regarding thoughts of suicide determined if the student was in the low suicide risk group. These students were not interviewed individually, but were offered participation in a guided internet-based cognitive behavioural therapy (CBT) program.

After screening, the results were rapidly available. The first variable examined was suicidal risk. From the responses to the three questions, students were placed in a category of none, low, medium, or high suicide-risk (Figs 2 and 3). The study staff identified the students at greatest risk based on this algorithm (those in the high suicide risk group and medium suicide risk group). *A priori* we determined that we would consider those individuals who were either in the high suicide risk group or the medium suicide group to comprise those who were “actively suicidal”. These students were then seen for an interview by a trained member of staff (usually somebody familiar to them) for a 1-hour semi-structured interview, which included a more detailed assessment of suicide risk and an open discussion of issues that were relevant to the individual student. Immediately following these interviews the parents (or guardian) was contacted and informed about the findings and concerns regarding the safety of that student. At this time a safety plan was agreed upon, including potential referral to Primary Care, Emergency, or specialized Mental Health services, as well as having information provided about the subsequent plans (to offer a guided internet approach with CBT). An informed consent sheet was provided to the student and parent/guardian regarding the guided CBT being offered, and both written parental and student consent were required to take part. Unfortunately, because of time and staffing constraints, each parent/guardian and the student only had 5 working days to return signed informed consent sheets to be eligible for the additional CBT programs.

Each of the three suicide-risk groups was treated differently. All those who were in the high suicide risk group were interviewed within 2 working days (but in over 90%, this happened within 24 hours of completion of the screening). For those in the medium suicide risk group, the same process occurred, but they were seen with within 5 working days (Fig 2). If they were in the low suicide risk group (Fig 3), they were not interviewed but were offered the opportunity to take part in one of two guided internet-based CBT programs. The first was focused on depression and was used by over 95% of students who took part (THIS WAY UP) [57]. The second program was offered to those High Risk students who had addiction issues (Breaking Free) [58], but very few students chose to utilize this. Both groups were offered as many supervised sessions as they required to complete these programs, although the actual number each student completed was not recorded in this pilot

#### Identification of high risk group (Top 10%)

In addition to those who had a suicide risk of any type (all of whom were offered the opportunity to take part in THIS WAY UP), we also identified the top 10% of those who had the highest overall scores on the combined EMPATHY scale score (see below). This was based on previous studies suggesting that up to 10% of students aged 10–18 may have significant symptoms of depression [79], [80]. Additionally, there is strong evidence that those who have “sub-threshold” depression are at raised risk of self-harm and suicide attempts [8], [46], [47]. It is also recognized that depression in youth is frequently associated with poor social performance, social withdrawal, increased use of drugs and alcohol, and low self-esteem [2]. For these reasons we determined that it may be important to intervene in those who may be at high-risk, as determined by these factors, and not simply by their scores on a depression scale. For this reason we determined that is this pilot examination of the EMPATHY program we would consider students to be in the High Risk group either if they were actively suicidal or if they scored in the top 10% for combined scores for their specific grade. It was not clear from previous research how much overlap there would be between the groups who had a raised suicide risk and those who were not actively suicidal but had a large number of other symptoms. It should be recognized firstly that that we recognized that a combination of similar scores has not been previously used to determine risk, and secondly that determination of 10% of those with the highest scores belonging in the High Risk group was arbitrary, and was not backed by evidence. We have *a priori* planned to carry out subsequent analyses to compare outcomes using more standard measures (such as depression scale scores alone, and using a different percentage for those at risk). This will allow us to make more evidence-based decisions in the future. We were also uncertain how many students in the High Risk group who were offered the guided CBT programs would take part, as previous research has suggested that many would not [50].

#### Combined EMPATHY scale score

Other studies have demonstrated that there is a large “invisible” group of youth who are at risk [8], and we planned to determine if the use of multiple indicators may help identify them. For this reason we created a summary score composed of information from all 5 of the scales utilized in screening (Table 2). In doing so, equal weight (20%) was given to each of the 5 scales being used in determining those at highest risk. This was because there is not sufficient evidence to reliably suggest the relative importance that should be given to the role of low self-esteem, the use of DAT, or the presence of anxiety in the determination of risk for subsequent suicide. While it may be argued that greatest importance should be given to scores on the depression scale, we had already identified those who were actively suicidal and felt there is not currently enough evidence to determine how much additional importance should be given to the other measures. By using a composite score (the “combined EMPATHY scale score”), which includes all 5 areas being studied, we avoided risking missing a key component, but retained the ability to subsequently determine which questions (listed in Table 2) were most useful in determining outcomes, as well as the correlations between each scale. Thus, each scale was scored with every question having equal weight. The score for each scale was then “adjusted” to a maximum of 20. For example, if the original score on the self-esteem scale (Table 2) was 15 out of a possible 30, then the “adjusted” score for the student was 10 out of 20. The “adjusted” scores for each of the scales were then totaled to provide a score out of potential maximum of 100, and this was termed the “combined EMPATHY scale score”. The combined EMPATHY scale score was therefore calculated by adding the various scores, adjusted as follows: raw depression score x 20/33 + raw anxiety score x 20/21 + raw score for DAT x 20/11 + [20 –(raw self-esteem score x 20/30)] + [20 –(raw quality-of-life score x 20/44)]. Note that higher raw scores for depression, anxiety, and DAT indicated more problems, whereas for self-esteem and quality-of-life higher raw scores indicated better outcomes, which is why the scores for the latter two were adjusted appropriately in the combined EMPATHY scale score calculation.

From the above calculations the 10% of students with the highest combined EMPATHY scale scores in each Grade were identified, with a goal that they would be interviewed within 14 days of screening, and their parent/guardian would also be contacted (in the identical manner as those who were actively suicidal). They were also offered the opportunity to take part in guided internet-based CBT programs.

#### Universal intervention—OVK

All students in Grades 7 and 8 received a modified version of a CBT program designed to reduce depressive symptoms, OVK [26–29]. It is usually given as a set of 16 sessions, each during a 45-minute classroom lesson period. The first 8 sessions are focused on CBT principles and the second 8 sessions are focused on social and educational learning. However, for reasons of time availability in this pilot version of EMPATHY only the first 8 sessions, i.e. those on CBT, were given. Given this, we were aware that any findings could not be compared to previous use of this program. It was administered by the Resiliency Coaches who received a 3-day training course on this program.

#### Statistical analysis

The primary statistical method was a paired design, in which each student who completed both baseline ratings and follow-up ratings was their own control. As the data showed evidence of non-normality, a non-parametric test was carried out to compare the differences between the mean scores at baseline and 12-week follow-up. This involved Wilcoxon signed-rank test (paired), unless otherwise specified, which is a non-parametric statistical test for testing hypotheses on medians. Although not shown in the present publication, median values for all items are available upon request to the corresponding author.

Statistical analysis was carried out on an “intention to treat” basis utilizing R, version 3.1.0 (R Foundation for Statistical Computing, Vienna, Austria) and Stata/IC 13.1 for Windows (StataCorp, College Station, Texas, USA). Correlations were calculated using IBM SPSS Statistics 20.0 (Chicago, Illinois, USA).

The study was designed to determine potential changes in depression scale scores and in suicidality. From previous studies, it was determined that there may be a 5% decrease in both these scores. To detect a statistically significant reduction of this size would require a sample size of 65 students in a 1-sided sample size calculation completed using the G Power calculator, version 3.1, with α = 0.05. Given our sample size of 3,500 students, of which we estimated a minimum of 4% (i.e. 140 students) would be depressed with a similar number being suicidal, this study was adequately powered.

## Results

The study was carried out in 5 schools in the Red Deer Public School system—all 3 of the Middle Schools (Grades 6, 7, and 8—average age 12.3 years, range 10–14) and both High Schools (Grades 9, 10, 11, and 12—average age 15.7 years, range 13–19). In the first screen (baseline), a total of 3,244 students took part (Fig 1), of whom 1,568 were female and 1,676 were male. In the second screen, a total of 3,228 students took part, of whom 1,550 were female and 1,678 were male. The total study population was 3,682 (Fig 1) although, only 2,790 students took part in both the baseline and 12-week follow-up assessment (i.e. in addition to the 2,790 students who completed both assessments, there were 454 students who only completed baseline assessments and 438 who only completed the follow-up assessments). Unless otherwise stated, all of the results reported are on the sample of 2,790 who completed both assessments, and were their own controls. Both mean ± standard deviation are shown.

### High risk groups

As previously noted, in the present piloting of the EMPATHY program we arbitrarily determined that we would include both those who were actively suicidal, as well as those who were in the top 10% of combined scores, as being in the High Risk group. Of the total 3,244 screened at baseline, 503 students were identified as being in the High Risk group, mostly because they had some suicidal symptoms (n = 378), of whom 220 (58%) also scored in the top 10% of combined scores. In the present study there were an additional 125 students who did not have significant suicidal symptoms but who scored highly on other ratings and were included in the High Risk group.

#### Combined EMPATHY scale scores

The combined EMPATHY scale scores represented overall scores based on all of the 5 measurements made, and decreased from a mean of 25.96 ± 15.05 to 23.99 ± 14.81 (an 8% decrease) which was highly statistically significant (p<0.0001) using Wilcoxon signed-rank test (paired). There were statistically significant differences seen in all but Grade 8, and in 4 of the 5 schools taking part in the study (Table 3).

**Table 3:** Variation by Grade and by School: change from baseline to 12-week follow-up.

	Grades, or number of students (for schools)	Mean Age at Baseline	dEMPATHY Scores statistical significance ^a^	Depression Scores statistical significance ^a^	Anxiety Scores statistical significance ^a^	Drugs, Alcohol, Tobacco Scores statistical significance ^a^	Self-Esteem Scores statistical significance ^a^	Quality of Life Scores statistical significance ^a^
**Variation by Grade**								
Middle School								
	6	11.25 ± 0.34	**p < 0.0001**	**p < 0.0001**	**p < 0.0001**	p = 0.86	**p < 0.0001**	**p < 0.0001**
	7	12.31 ± 0.35	**p < 0.01**	**p < 0.02**	**p < 0.05**	p = 0.05	p = 0.21	p = 0.48
	8	13.31 ± 0.37	p = 0.10	**p < 0.02**	**p < 0.05**	p = 0.21	p = 0.74	p = 0.71
High School								
	9	14.31 ± 0.35	**p < 0.01**	**p < 0.01**	**p < 0.001**	p = 0.10	**p < 0.05**	p = 0.68
	10	15.30 ± 0.36	**p < 0.0001**	**p < 0.001**	**p < 0.0001**	p = 0.94	**p < 0.0001**	p = 0.12
	11	16.35 ± 0.38	**p < 0.001**	**p < 0.001**	**p < 0.001**	p = 0.89	**p < 0.01**	p = 0.22
	12	17.39 ± 0.54	**p < 0.0001**	**p < 0.0001**	**p < 0.0001**	p = 0.08	**p < 0.0001**	**p < 0.0001**
**Variation by School**								
Middle School 1 (Grades 6, 7, 8)	321	12.26 ± 0.89	p = 0.15	**p < 0.02**	**p < 0.01**	p = 0.89	p = 0.10	p = 0.09
Middle School 2 (Grades 6, 7, 8)	504	12.26 ± 0.91	**p < 0.0001**	**p < 0.0001**	**p < 0.0001**	p = 0.56	**p < 0.0001**	**p < 0.001**
Middle School 3 (Grades 6, 7, 8)	280	12.31 ± 0.88	**p < 0.01**	**p < 0.01**	**p < 0.05**	p = 0.24	**p < 0.001**	**p < 0.05**
High School 1 (Grades 9, 10, 11, 12)	786	15.65 ± 1.16	**p < 0.0001**	**p < 0.0001**	**p < 0.0001**	p = 0.94	**p < 0.0001**	**p < 0.05**
High School 2 (Grades 9, 10, 11, 12)	899	15.67 ± 1.18	**p < 0.0001**	**p < 0.0001**	**p < 0.0001**	p = 0.82	**p < 0.0001**	**p < 0.01**

^a^ Statistical significance using Wilcoxon signed-rank test (paired). Bold type indicates statistical significance.

In terms of correlations between each of the subscales of the combined EMPATHY scale score, the depression, anxiety, self-esteem, and quality of life scores were all significantly correlated, although the DAT score was only weakly significantly correlated with the quality-of-life score but no other scales (Table 4).

**Table 4:** Correlations between the scores on the 5 subscales in the combined EMPATHY scale score, using Spearman’s rank correlation coefficient.

	Depression scores	Anxiety scores	Drugs, Alcohol, Tobacco scores	Self-esteem scores	Quality-of-life scores
**Depression scores**	1.000	**0.684**	0.289	**-0.693**	**-0.726**
**Anxiety scores**	**0.684**	1.000	0.214	**-0.637**	**-0.659**
**Drugs, Alcohol, Tobacco scores**	0.289	0.214	1.000	-0.210	-0.241
**Self-esteem scores**	**-0.693**	**-0.637**	-0.210	1.000	**0.756**
**Quality-of-life scores**	**-0.726**	**-0.659**	-0.241	**0.756**	1.000

In addition to examining changes in the group who consisted of the highest 10% of scores on the combined EMPATHY scale score, we also examined the group who comprised the top 25% of combined EMPATHY scale scores (n = 698) (Table 5). In this group of more symptomatic students, there was a highly significant reduction in the combined EMPATHY scale score (13.4%), depression score (28.9%), and anxiety score (17.7%). There were also small, but statistically significant, decreases in the scores for the use of DAT, self-esteem, and quality-of-life (Table 5).

**Table 5:** Change in scores from baseline to follow-up for those scoring in top 25% of combined EMPATHY scale scores.

	Students scoring in top 25% of combined EMPATHY scale scores		
	Pre-intervention mean (SD)	Post-intervention mean (SD)	Statistical significance ^a^
**Combined EMPATHY scale score**	47.10 ± 10.16	40.81 ± 13.98	**p<0.0001**
**Depression score**	9.65 ± 3.31	6.86 ± 4.32	**p<0.0001**
**Anxiety score**	13.96 ± 2.27	11.49 ± 4.35	**p<0.0001**
**Drugs, Alcohol, Tobacco score**	7.65 ± 3.92	6.80 ± 4.51	**p<0.0001**
**Self-Esteem score**	11.67 ± 1.62	10.31 ± 2.86	**p<0.0001**
**Quality of Life score**	11.14 ± 1.76	9.85 ± 2.88	**p<0.0001**
	**Students who took the guided internet-based cognitive behavioural therapy (CBT) intervention ± SD (n = 151)**		
**Combined EMPATHY scale score**	52.84 ± 10.17	45.23 ± 15.86	**p<0.0001**
**Depression score**	11.52 ± 3.71	8.34 ± 4.91	**p<0.0001**
**Anxiety score**	14.10 ± 3.54	12.39 ± 4.80	**p<0.0001**
**Drugs, Alcohol, Tobacco score**	4.50 ± 5.92	4.06 ± 5.86	p = 0.15
**Self-Esteem score**	11.99 ± 2.32	10.87 ± 3.02	**p<0.0001**
**Quality of Life score**	11.31 ± 2.41	10.15 ± 3.35	**p<0.0001**

^a^ Statistical significance using Wilcoxon signed-rank test (paired). Bold type indicates statistical significance.

#### Impact of guided internet-based CBT interventions

**Fig 4:**
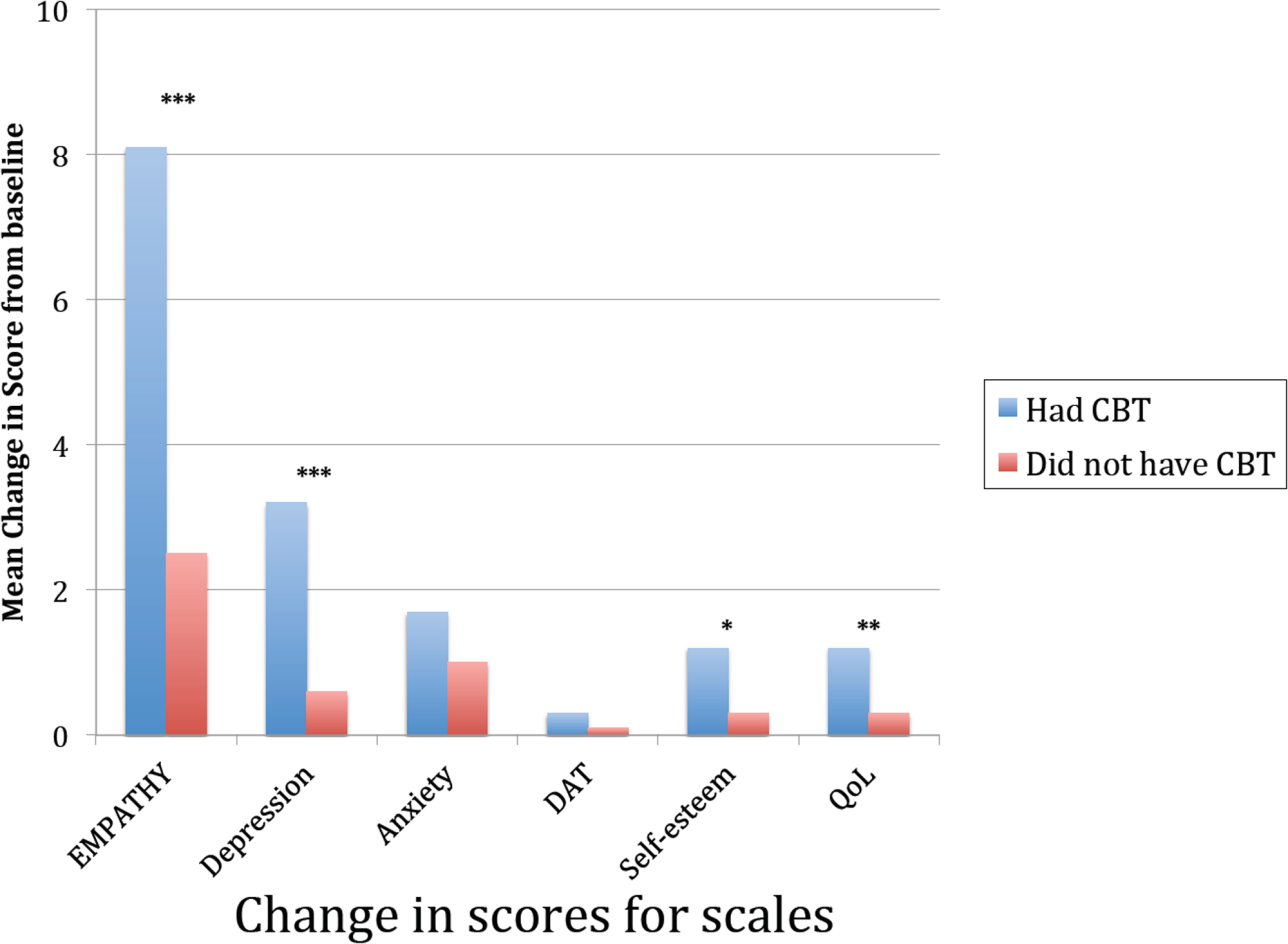
Difference in outcome in High Risk group depending if they had guided internet-based CBT. There were a total of 409 individuals who were identified as being in the High Risk group who had ratings at both baseline and at 12-week follow-up. Of these 151 took part in the guided internet-based cognitive behavioural therapy (CBT) program. The figure shows the mean change in score for the EMPATHY scale and each of the 5 subscales (depression, anxiety, drugs, alcohol, and tobacco (DAT), self-esteem, and quality-of-life (QoL)) for the 151 who took part compared to changes in score in the 258 who did not. It can be seen that the group who took part had significantly better reductions in EMPATHY and depression scores. There were also smaller, but significant, reductions in self-esteem and quality of life in the group who had the CBT program. The following symbols indicate the degree of statistical significance, * p<0.05, ** p<0.001, *** p<0.0001

A total of 503 students were offered guided internet-based CBT interventions. Of these, 151 students took part (30%), with over 90% completing THIS WAY UP (focused on depression). There were highly significant improvements compared to baseline with deceases in the combined EMPATHY scale score (a 14% decrease), depression scores (a 28% decrease), and anxiety scores (a 12% decrease) (Table 5). There were also small, but statistically significant decreases in scores for self-esteem and quality-of-life. Importantly, the mean change from baseline for both combined EMPATHY scale score and depression score in the 151 who took part in the CBT treatment was significantly greater than in the other members of the High Risk group who did not take part in the guided internet-based CBT treatment programs (n = 352) (Fig 4). There were no statistically significant differences between these two groups in terms of age, sex, or any baseline scores on any of the measures, and there were no statistically significant differences (p = 0.52) in those who responded and were in Middle Schools (Grades 6, 7, and 8: n = 87) and those who were in High Schools (Grades 9, 10, 11, and 12: n = 64).

### Depression scores

**Fig 5:**
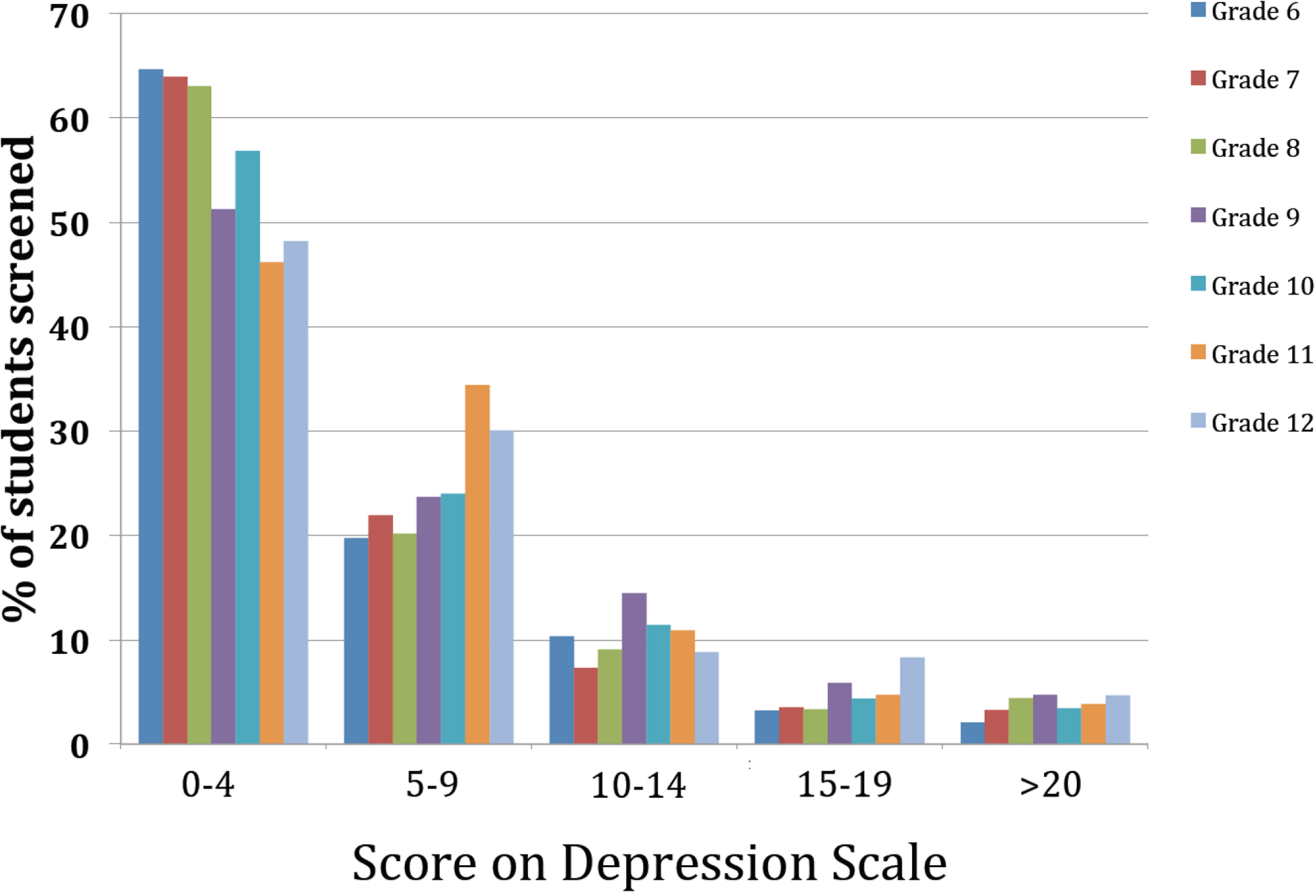
Distribution of depression scores at baseline. The distribution of depression scores is shown for all 7 Grades (average ages at start of study are 11.3 for Grade 6, 12.3 for Grade 7, 13.3 for Grade 8, 14.3 for Grade 9, 15.3 for Grade 10, 16.4 for Grade 11, and 17.4 for Grade 12). Although not directly comparable to previous studies, as the depression score was not directly comparable to PHQ-A scores, and do not have diagnostic validity, we indicate the distribution of students into the ranges that in previous PHQ-A studies have been proposed to indicate the presence of a depressive disorder. These are not depressed (0–4), minor symptoms (5–9), mild depression (10–14), moderate depression (15–19), and severe depression (>20). It can be seen that the distribution of symptoms shows that scores compatible with mild depression are seen most frequently in Grade 9, whereas scores compatible with severe depression occur relatively equally across all Grades from 7–12.

At baseline, the scores for the total of 3,244 students who completed the depression scales varied by Grade (Fig 5). The scores for depression were grouped according to ranges that have previously been used for indication of possible diagnosis of depressive disorder, although it is recognized that with the changes from the original PHQ-A these results are not directly comparable. The ranges used in previous studies are a score of 0–4 indicating not depressed, a score of 5–9 indicating the presence of minor symptoms, a score of 10–14 which may indicate the presence of a mild depressive disorder, a score of 15–19 which may indicate a moderate depressive disorder, and a score of 20 or more which may indicate the presence of severe depression. In the present study, it can be seen that the distribution of scores varies across the Grades. Scores compatible with mild depression are seen most frequently in Grade 9, whereas scores compatible with severe depression occurred relatively equally across all Grades from 7–12 (Fig 5).

Comparing baseline ratings in the 2,790 students who completed both measurements to those at the 12-week follow-up, the mean total depression score decreased significantly from 3.71 ± 3.86 to 3.17 ± 3.51 (a 15% decrease), which was highly statistically significant (p<0.0001 using Wilcoxon signed-rank test for paired samples). There were statistically significant reductions in all Grades and in all schools (Table 3).

#### Suicidality scores

For the 2,790 students who completed both baseline and follow-up ratings at 12-weeks, there was a marked reduction in the number of students who were actively suicidal, which we had *a priori* defined as those who were in either the high suicide risk group or the medium suicide risk group, from 125 at baseline to 30 at follow-up (Table 6). There were also reductions in the assessed level of suicide risk for many of these students, and out of 71 students who were in the high suicide risk group before the intervention, 41 of them transitioned to having no suicide risk at follow-up, while out of the 54 students in the medium suicide risk group at baseline, 35 transitioned to the no suicide risk group at follow-up (Table 6).

**Table 6:** Level of suicide risk at 12-week follow-up in students who completed both ratings (n = 2,790).

	High suicide risk at 12-weeks follow-up	Medium suicide risk at 12-weeks follow-up	Low suicide risk at 12-weeks follow-up	No suicide risk at 12-weeks follow-up
**Level of risk at baseline**				
**High suicide risk (n = 71)**	13	5	12	41
**Medium suicide risk (n = 54)**	6	6	7	35
*Students “actively suicidal”(High + Medium suicide risk) at baseline*	(125)			
*Students “actively suicidal”(High + Medium suicide risk) at 12-week follow-up*	(30)			
**Low suicide risk (n = 79)**	13	2	13	51
**Totals (n = 204)**	32	13	32	127

In addition to the analysis of students who completed both ratings, there was also a smaller reduction in the numbers of students who were actively suicidal in the total student group. Thus, at the baseline rating among the 3,244 students a total of 125 were actively suicidal compared to 104 students who were actively suicidal at the 12-week follow-up completed by 3,228 students (Table 7).

**Table 7:** Number of students in different risk levels for suicide at baseline and at 12-week follow-up for total population.

	Baseline (sample size n = 3,244)	At 12-week follow-up (sample size n = 3,228)
**Level of risk at baseline**		
**High suicide risk**	71	64
**Medium suicide risk)**	54	40
*Students “actively suicidal”(High + Medium suicide risk)*	*(125)*	*(104)*
**Low suicide risk**	79	86
**Totals**	204	190

### Anxiety scores

**Fig 6:**
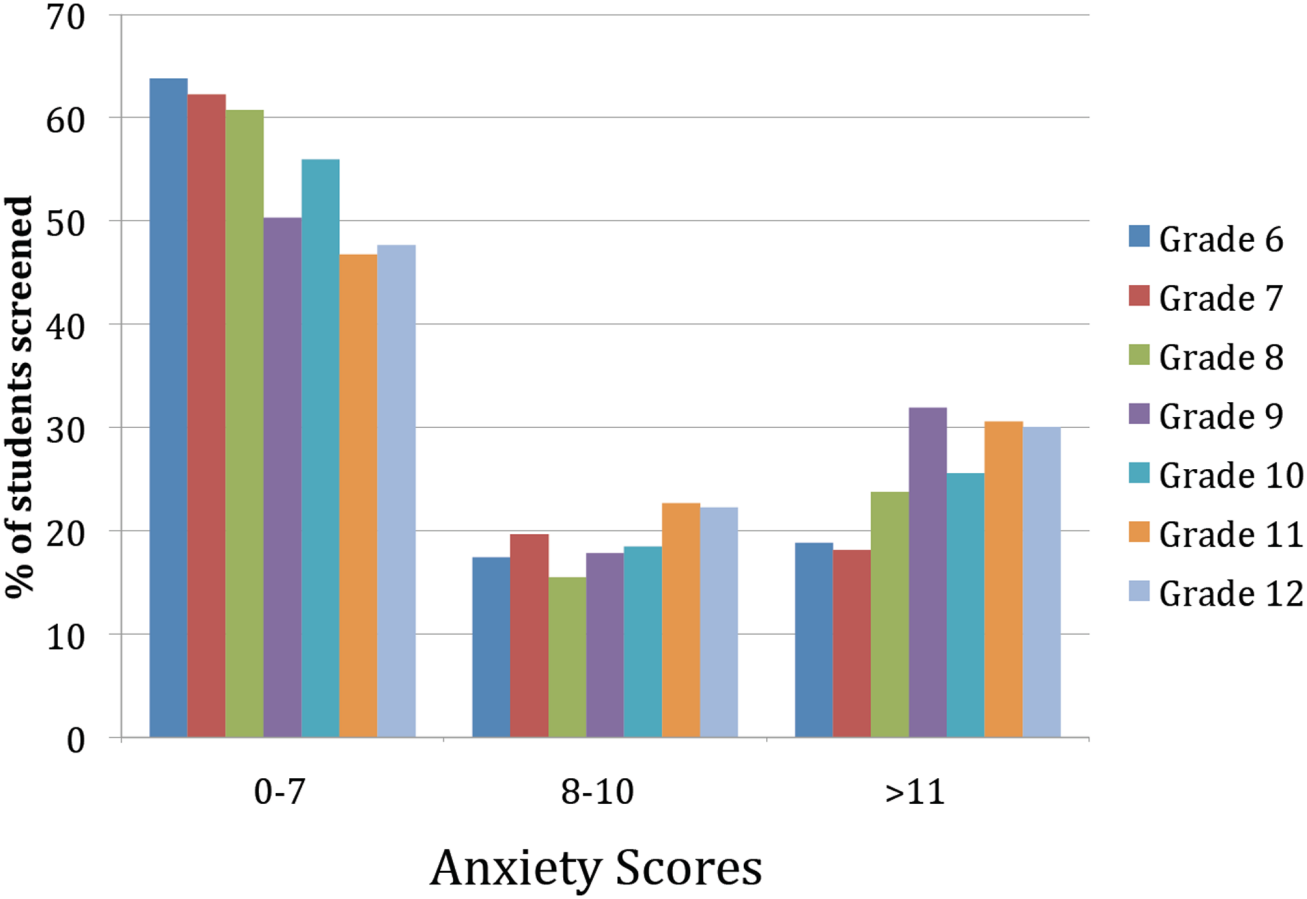
Distribution of anxiety scores at baseline. The distribution of anxiety scores is shown for all 7 Grades (average ages at start of study are 11.3 for Grade 6, 12.3 for Grade 7, 13.3 for Grade 8, 14.3 for Grade 9, 15.3 for Grade 10, 16.4 for Grade 11, and 17.4 for Grade 12). While these scores do not have diagnostic validity, we indicate the distribution of anxiety scores that in previous studies have been proposed to indicate the presence of an anxiety disorder. These are not anxious (0–7), possible anxiety disorder (8–10), and anxiety disorder (>11). It can be seen that there more than 30% of students in Grades 9, 11, and 12 how scored 11 or more, suggesting frequent anxiety symptoms. Even in the younger Grades more than 15% of students in every grade had frequent anxiety symptoms. This finding is consistent with previous research regarding the frequency of anxiety disorders in youth.

At baseline, the scores for the total of 3,244 students varied by Grade (Fig 6). It can be seen that more than 30% of students in Grades 9, 11, and 12 had a significant number of anxiety symptoms, but even in younger Grades more than 15% of students in every grade had frequent symptoms of anxiety. This finding is consistent with previous research regarding the frequency of anxiety symptoms in youth.

Comparing baseline ratings in the 2,790 students who completed both measurements to those at the 12-week follow-up, the mean total anxiety scores decreased significantly from 6.97 ± 4.72 to 6.22 ± 4.83 (an 11% decrease), which was highly statistically significant (p<0.0001 using Wilcoxon signed-rank test for paired samples). There were statistically significant differences in all Grades and in all schools (Table 3).

### Drugs, alcohol, and tobacco (DAT) scores

**Fig 7:**
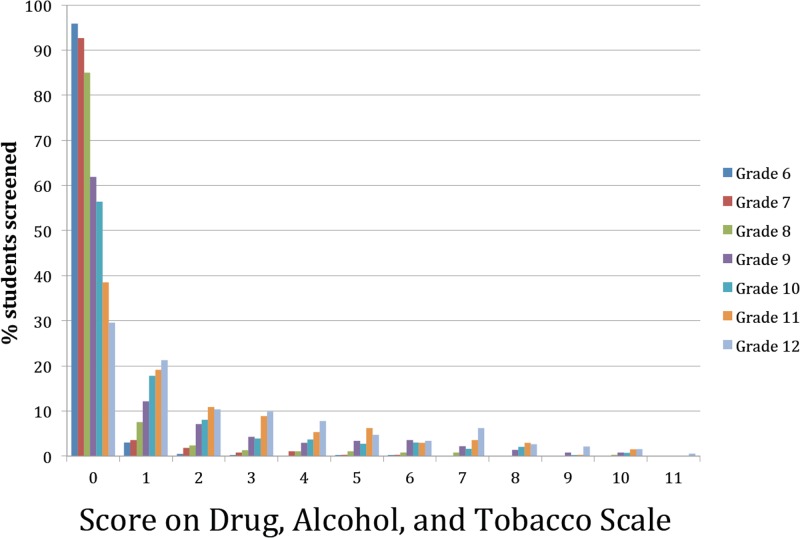
Distribution of drugs, alcohol, and tobacco scores at baseline. The distribution of scores for the use of drugs, alcohol, and tobacco is shown for all 7 Grades (average ages at start of study are 11.3 for Grade 6, 12.3 for Grade 7, 13.3 for Grade 8, 14.3 for Grade 9, 15.3 for Grade 10, 16.4 for Grade 11, and 17.4 for Grade 12). While these scores do not have diagnostic validity, we indicate the distribution of these scores. It can be seen that at all scores the distribution is that the oldest youth have more use of drugs, alcohol, and tobacco, and have been involved in more significant activities that may put them at risk. It is consistent with previous studies that less than 30% of youth in Grade 12 have not used drugs, alcohol, or tobacco in the previous 12-months, compared to more than 95% of those in Grade 6.

At baseline, the scores for the total of 3,244 students who completed DAT scales varied widely by Grade, with only 29% of the Grade 12 students never having abused DAT compared to 95% of the Grade 6 students (Fig 7). It can also be seen that there was a consistent relationship between Grade (and age) and use of DAT, with the greatest use being in the higher Grades (i.e. older age groups).

In marked contrast to the findings for depression and anxiety, when comparing baseline ratings in the 2,790 students who completed both measurements to those at the 12-week follow-up, there no statistically differences between the mean score at baseline (mean 1.90 ± 3.59) compared to that at 12-weeks (mean 1.86 ± 3.55) (p = 0.92). There were also no statistically significant changes seen across any of the school Grades, or in any of the schools (Table 3).

### Self-esteem scores

Comparing baseline ratings to those at the 12-week follow-up there was a statically significant decrease in mean self-esteem scores. Comparing baseline ratings to those at the 12-week follow-up in the 2,790 students who completed both measurements there was a small (5.0%) decrease in mean total self-esteem scores from 7.32 ± 3.18 to 6.95 ± 3.25, which was statistically significant (p<0.0001 using Wilcoxon signed-rank test for paired samples). Given the improvement in depression and anxiety scores, this was unanticipated. This decrease in self-esteem was seen across most Grades and in 4 of the 5 schools (Table 3).

### Quality-of-life scores

Similar to the findings for self-esteem, comparing baseline ratings to those at the 12-week follow-up in the 2,790 students who completed both measurements, there was a statically significant decrease in mean quality-of-life scores. Comparing baseline ratings to those at the 12-week follow-up, there was a small (3.6%) decrease in mean total self-esteem scores from 6.39 ± 3.36 to 6.16 ± 3.45, which was nonetheless statistically significant (p<0.0001 using Wilcoxon signed-rank test for paired samples). Given the improvement in depression and anxiety scores, again this finding was unanticipated. This decrease in quality-of-life score was only seen in two Grades but in 4 of the 5 schools (Table 3).

### Role of OVK

In the study, two Grades, 7 and 8 (mean ages at the start of the study for students in these Grades were 12.3 and 13.3, respectively), also received a shortened version of OVK. In trying to determine whether this had a significant impact upon the depression scores, we found no differences in the mean change in depression scores between these two grades (n = 737) and those of either Grade 6 students (n = 368) or Grade 9 students (n = 460). For example, the mean decrease in the depression score for Grade 7 and 8 students who had OVK was 0.36 ± 3.1, whereas for those in Grade 9, it was 0.52 ± 3.1 (p = 0.66). These findings do not support our initial supposition that the addition of the modified OVK program would lead to further additional improvements in depression scores. However, as previously noted, given that this was only a limited version of the usual scale this finding should not be used to consider the effectiveness, or otherwise, of the OVK intervention. Further studies using the full scale are required to determine this in a similar study population.

## Discussion

Our primary hypothesis was that this approach would reduce both depression and suicidality, and our secondary hypotheses were that this program would also reduce anxiety and use of DAT while increasing self-esteem and quality-of-life. While our primary hypotheses was supported, many of the secondary hypotheses were not.

The results from the present study show a highly significant decrease in depression scores (approximately 15%), seen in all age groups and in all schools, and the impact appeared greater in those who had more symptoms at baseline. There were also significant decreases in anxiety scores in all Grades and in all schools. We also identified a High Risk group, who were those students who were either suicidal and/or were in the top 10% of total EMPATHY scores in each individual school. Those in the High Risk who took part in subsequent guided internet-based CBT programs did better than individuals with a similar level of severity who did not. There appeared no baseline differences between these two groups, but they were not randomized into these two groups so it is possible that this finding is due to other, currently unclear, reasons.

Possibly more importantly, there were significant reductions in the number of students who were actively suicidal. Thus, in the actively suicidal students at baseline who completed both assessments, 76 (61%) of them were in the no-risk group at the end of the 12-week follow-up period, while the number in the actively suicidal group (those in either the high risk suicide group or the medium risk suicide group) decreased from 125 to 30.

There were no significant changes in the use of DAT scores. However, this latter finding was not surprising as the questions relate to use over the previous 12-months and the follow-up was only during the previous 3-months. Longer-term follow-up will be needed to determine if there are any DAT changes. The total combined EMPATHY scale score also decreased significantly in many schools and Grades, and with the high level of correlation between all sub-scales (apart from the DAT scores), this might support the use of such an aggregate score in future studies.

In terms of the design of this study, we used a multimodal approach incorporating several components, each of which individually has been reported as having some benefits towards improving depression. Additionally, we utilized technology whenever possible and this allowed ultra-rapid identification of the high-risk group. We also carried out the program in a complete geographical region, in contrast to many previous studies which have sometimes included schools which self-selected to be involved. It is therefore clear that the EMPATHY program is somewhat different to those previously studied.

Caution in terms of the sustainability of the improvements seen in depression is required, as other studies have previously shown improvements over a 3-month period that are not sustained over longer time periods [81]. In terms of a more comprehensive approach, the study, which is most similar to the EMPATHY program, is a program carried out in Australia containing many of the same elements [50]. Unfortunately, this large and well-designed study found no improvements in depression over a 3-year period. This emphasizes the need for a longer-term follow-up.

If these initial positive findings are confirmed during longer-term follow-up study then, since many different and well-designed studies have failed to find similar positive outcomes, what are the key potential reasons that the present study has had such a positive effect on many measures? We believe that there are several key components that may have contributed. The first may have been the very rapid feedback from the time students completed their screen until the time that they were contacted (less than 48 hours if they were in the high suicide risk group). Feedback from individual students was that this was very helpful, as the rapid feedback made them feel that their issues were taken seriously. Supporting this possibility, a recent qualitative investigation of adolescents’ perceived mechanism of change following a universal school-based depression program found that there were improved interpersonal relationships and improved self-regulation, both stronger than originally assumed [82]. Additionally, the fact that parent/guardian became rapidly involved, having frequently not been aware of the severity of the issue, may also have been a contributing factor to an improvement in mood, anxiety, and suicidal thinking by students [83], [84]. Supporting the hypothesis that parent/guardian attitudes may have changed was the fact that during the study there were extremely frequent positive comments from parent/guardian expressing gratitude about knowing this information about their child.

A second possible factor could have been a change in the overall school environment or culture. While we did not formally study this, such changes have been reported in school environments previously [85], and changes in school environments can improve student mental health [86]. The possibility that this occurred was suggested by frequent anecdotal feedback from principals, teachers, students, and parents. However, to determine if this occurred, it requires specific future study.

A third possibility that may have had an impact was that this program was carried out across a complete school district, as opposed to single schools. It is conceivable that this had the effect of heightening community awareness of, and support for, the program. It was certainly clear that children at different schools were in frequent contact about the program for a variety of reasons, including that many children had elder or younger siblings at other schools. There was also significant local media interest in the EMPATHY program. It is well established that community attitudes can be important in determining frequency of depression in youth [87]. It is therefore conceivable that a positive change, or awareness regarding this issue, may have been one factor in the changes seen in the present study.

Fourthly, the Resiliency Coaches spent a lot of time with the students as part of the staff, including during some student recreation time, and although they were specifically trained to not act as “therapists”, they found that on many occasions students would spontaneously approach them and sometimes confide in them (including about physical and/or sexual abuse they had experienced or witnessed). The impact of this is uncertain, but this is a different role to that held by trained psychologists working at the school (as well as by the teachers, of course), and the Resiliency Coaches were surprised by how frequently their availability allowed such spontaneous interactions to occur. It is recognized that friendship skills can help adolescent depressive symptoms in combination with other approaches [88], so it is possible this may also have played a role. Currently, however, these four possibilities remain speculative. In future studies it will be important to examine them more specifically to determine what, if any, impact each of these has.

In terms of the potential benefit for a universal CBT intervention (OVK) this remains uncertain. Given that in the present study it was used in a different and truncated manner, and administered by a different group of individuals, than those who have previously been shown to deliver it effectively, it cannot be considered to have been adequately tested in the present study. That universal interventions may be more effective when delivered by psychologists has been suggested from other studies [89], means that we are uncertain what our negative findings may indicate. Determining the relative importance of this aspect of the program will be required in future studies, and most importantly it is necessary to study the effectiveness of the full 16-session program. Nonetheless, it is well recognized that it can be difficult to detect the overall effect of such universal interventions, and this may explain the large number of studies that are unable to show a benefit. The potential benefit of universal approaches such as OVK, as well as those recently examined for suicide prevention [9], [52], needs to be evaluated as part of the approach of combination programs such as EMPATHY, but are unlikely to be measures that on their own significantly reduce youth depression and suicide rates.

Unexpectedly, there were small, but statistically significant, decreases in both self-esteem and quality of life-scores (5.0% and 3.6% respectively). While the data demonstrated the expected findings that at baseline in those who had higher depression scores there was a close correlation with lower self-esteem and quality-of-life scores, the small decrease in both of these after the 12-week study period was unanticipated. Currently, we do not have a clear reason why this may have occurred, and although we believe the size of these changes means they were not clinically relevant, the fact that they appeared in several Grades means it will be important to continue to measure these factors in future studies. We also plan to carry out more detailed qualitative research to try and determine why this may have occurred.

### Potential issues with the study

There were three major potential limitations of the present study, the first being that it was a short-term study, and it is uncertain if the changes identified will be sustained. It could be argued that in terms of suicidality, reducing this even for short periods may reduce the risks of completed suicide. Nonetheless, it is recognized that the links between scores for suicidality on a rating scale and actual suicide attempts, and in turn the number of completed suicides, remains uncertain. Thus, while there was a very gratifying reduction in suicidality ratings, it is not clear that this will translate into fewer episodes of self-harm or a reduction in completed suicides. What gives us some hope that this will be the case is that in about half of the students who were identified as being at high risk (either because of suicidal thoughts, or for high combined EMPATHY scale scores) they had not previously been recognized as being a concern either to their parent/guardian or to the school staff. This group may therefore have benefited significantly from recognition. Also, there is good evidence that youth who are suicidal usually show verbal, behavioural, and environmental warnings [90], [91], and so it is likely that the symptoms detected by the questions asked represent meaningful measurements of suicide risk. Our finding of a large group of high-risk children who were not previously identified is very consistent with data from a recent large study in over 12,000 adolescents, part of the Saving and Empowering Young Lives in Europe (SEYLE) project, which recruited these youth in randomly selected schools in 12 European countries [8]. This study found that there was a large group of high-risk students with active suicidal thoughts who were “invisible”, but whose risk of suicide attempts was 6%, and we believe that this may represent a significant proportion of students identified in the current study as being high suicide risk, even if their depression scores were not very raised. Given the high rates of actual attempts at suicide in this group, it is certainly possible that reducing this risk provides a meaningful reduction in the number of youth who will attempt suicide. It remains important, however, that longer-term follow-up is carried out to determine if the improvements in mood, anxiety, and suicidality detected after 12-weeks are retained over the longer-term.

The second major issue with this study is the lack of a robust control group, and not having such a control means any conclusions about the EMPATHY program should be somewhat cautious at this time. We are, therefore, unable to determine if there would have similar improvements without any intervention. Certainly other studies in similar populations have shown that there is frequently a decrease in depression symptoms, regardless of what treatment is used, and this can be no different from controls [49]. There were compelling ethical reasons why we did not have a control group of students (either at the same or different schools), because the most rigorous control group would have involved screening for active suicidality and then not intervening. Nonetheless, given that the improvements in depression, suicidality, and anxiety, occurred in all Grades and in all schools (which serve quite different populations), and not simply in motivated schools, we believe this unlikely although it will clearly require future additional research to determine the validity of this.

The third potential issue with the study was the use of the rating scales in a different manner to which they had designed or validated. We utilized a novel, and untested, approach to determining who was at risk. This was to combine several scales, each of which has been used independently, into a single score that was then used to determine who would receive additional intervention. Future research will need to determine the effectiveness of this approach in identifying those at highest-risk over the longer-time, and compare this to the use of an individual scale used its original format (such as PHQ-A). Such research will also clarify if the cut-off we determined (the top 10%) was appropriate, and whether any such cut-off is best applied across a Grade or across an entire school. Additionally, since many of the scales were utilized in a manner that differs from the method in which they have been previously validated (such as the additional suicide question added to the PHQ-A and the questions about tobacco added to the CRAAFT), research will need to clarify how each of the individual scales performed utilizing their original methodology. However, the high correlations between 4 of the 5 scales (all except those for the use of DAT) suggest that this approach may have some validity.

## Conclusions

In conclusion, the EMPATHY program appears to offer the potential for meaningful improvements in depression and anxiety in large number of youth, and a potential major reduction in the number of youth who are actively suicidal. Further research is required to determine if these findings are maintained in the longer-term, and if so, then what the most important aspects of the program are. It also remains to be seen if it can be successfully implemented in other jurisdictions.

## Supporting Information

S1 TREND Checklist This checklist details the information regarding whether or not methodological issues were addressed, and if so what page they can be seen. Note that the page numbering refers to the original manuscript submission, and will differ from any final published format.
(PDF)

S1 Protocol Original protocol with changes highlighted. This shows the original protocol approved by the ethics review committee in September 2013, as well as the subsequent changes made which were approved in January 2014. These changes relate in large degree to a change in the measurement tools, necessitated by a desire to utilize screening tools, and cognitive behavioural treatment (CBT) tools, that would fit within the paradigms established in the study, i.e. being free, brief, and available to use with the technology available to the study and the schools. It can be seen that there varied considerably from the initial tools that were approved for use.
(PDF)
