# Supplementary material for: Initial Findings from a Novel School-Based Program, EMPATHY, Which May Help Reduce Depression and Suicidality in Youth
Source: PLoS One. 2015 May 14;10(5):e0125527. doi: 10.1371/journal.pone.0125527 (PMC4431804; doi:10.1371/journal.pone.0125527)
Supplement: S1 TREND Checklist — Note that the page numbering refers to the original manuscript submission, and will differ from any final published format. (PDF) [file pone.0125527.s001.pdf]

TREND Statement Checklist

| Paper Section/ Topic | Item No | Descriptor | Reported? | |
|---|---|---|---|---|
| | | | ✔ | Page # |
| **Title and Abstract** | | | | |
| Title and Abstract | 1 | Information on how students allocated | ✔ | 2 |
| | | Structured abstract | ✔ | 2 |
| | | Information on target population | ✔ | 2 |
| **Introduction** | | | | |
| Background | 2 | Scientific background and explanation of rationale | ✔ | 3 |
| | | Theories used in designing behavioural interventions | ✔ | 3 |
| **Methods** | | | | |
| Participants | 3 | Eligibility criteria for participants | ✔ | 10 |
| | | Methods of recruitment | ✔ | 7 |
| | | Recruitment setting | ✔ | 8 |
| | | Setting and locations where data collected | ✔ | 8 |
| Interventions | 4 | Details of interventions intended for each study condition and how and when they were actually administered, including | ✔ | 12 |
| | | Content: what was given | ✔ | 10 |
| | | Delivery method: how was content given? | | 10 |
| | | Unit of delivery: how were the subjects grouped during delivery? | ✔ | 10 |
| | | Deliverer: who delivered the intervention? | ✔ | 10 |
| | | Setting: where was the intervention delivered? | ✔ | 8 |
| | | Exposure quantity and duration: how many sessions or episodes were intended to be delivered? How long were they intended to last? | ✔ | 10 |
| | | Time span: how long was it intended to take to deliver the intervention to each unit? | ✔ | 13 |
| | | Activities to increase compliance or adherence (e.g. incentives) | N/A | |
| Objectives | 5 | Specific objectives and hypotheses | ✔ | 6 |
| Objectives | 6 | Clearly defined primary and secondary outcome measures | ✔ | 6 |

| | | | | |
|---|---|---|---|---|
| | | Methods used to collect data and any methods used to enhance the quality of measurements | ✔ | 7 |
| | | Information on validated instruments such as psychometric and biometric properties | ✔ | 10 |
| Sample Size | 7 | How sample size was determined | ✔ | 16 |
| Assignment Method | 8 | Unit of assignment | ✔ | 16 |
| | | Methods used to assign units to study conditions, including details of any restriction | ✔ | 16 |
| | | Inclusion of aspects employed to minimize potential bias induced by non-randomization (e.g., matching) | N/A | |
| Blinding (masking) | 9 | Whether or not participants, those administering the interventions, and hose assessing the outcomes were blinded to the study condition assignment | N/A | |
| Unit of Analysis | 10 | Description of the smallest unit that is analyzed to assess intervention effects (e.g., individual, group, or community) | ✔ | 16 |
| | | If the unit of analysis differs from the unit of assignment, the analytical method used to account for this | N/A | |
| Statistical Methods | 11 | Statistical methods used to compare study groups for primary methods outcome(s) | ✔ | 16 |
| | | Statistical methods used for additional analyses, such as subgroup analyses and adjusted analysis | ✔ | 16 |
| | | Methods for imputing missing data, if used | N/A | |
| | | Statistical software or programs used | ✔ | 16 |
| **Results** | | | | |
| Participant flow | 12 | Flow of participants through each stage of the study: enrollment, assignment, allocation, and intervention exposure, follow-up, analysis | ✔ | 17 |
| | | Enrollment: the number screened for eligibility | ✔ | 17 |
| | | Assignment: the number assigned to a study condition | ✔ | 17 |
| | | Allocation and intervention exposure: the number of participants | ✔ | 17 |

| | | | | |
|---|---|---|---|---|
| | | assigned to each study condition and the number who received each intervention | | |
| | | Follow-up: the number of participants included in or excluded from the main analysis, by study condition | ✔ | 17 |
| | | Description of protocol deviations from study as planned, along with reasons | N/A | |
| Recruitment | 13 | Dates defining the periods of recruitment and follow-up | ✔ | 7 |
| Baseline Data | 14 | Baseline demographic and clinical characteristics of participants in each study condition | ✔ | 17 |
| | | Baseline characteristics for each study condition relevant to specific disease prevention research | ✔ | 17 |
| | | Baseline comparisons of those lost to follow-up and those retained, overall and by study condition | ✔ | 17 |
| | | Comparison between study population at baseline and target population of interest | N/A | |
| Baseline equivalence | 15 | Data on study group equivalence at baseline and statistical methods used to control for baseline differences | N/A | |
| Numbers analyzed | 16 | Number of participants (denominator) included in each analysis for each study condition, particularly when the denominators change for different outcomes | ✔ | 17 |
| | | Indication of whether the analysis strategy was "intention to treat" or, if not, description of how non-compliers were treated in the analysis | ✔ | 16 |
| Outcomes and estimation | 17 | For each primary and secondary outcome, a summary of results for each estimation study condition | ✔ | 17 |
| | | Inclusion of null and negative findings | ✔ | 17 |
| | | Inclusion of results from testing pre-specified causal pathways through which the intervention was intended to operate, if any | N/A | |
| Ancillary Analyses | 18 | Summary of other analyses performed, including sub-group or restricted | ✔ | 17 |

| | | | | |
|---|---|---|---|---|
| | | analyses, indicating which are pre-specified or exploratory | | |
| Adverse events | 19 | Summary of all important adverse events or unintended effects in each study condition | N/A | |
| **Discussion** | | | | |
| Interpretation | 20 | Interpretation of the results, taking into account study hypotheses, sources of potential bias, imprecision of measures, multiplicative analyses, and other limitations or weaknesses of the study | ✔ | 22 |
| | | Discussion of results taking into account the mechanism by which the intervention was intended to work | N/A | |
| | | Discussion of the success of and barriers to implementation of this intervention, fidelity of implementation | ✔ | 27 |
| | | Discussion of research, programmatic, or policy implications | ✔ | 22 |
| Generalizability | 21 | Generalizability (external validity) of trial findings, taking into account the study population, the characteristics of the intervention, length of follow-up, incentives, compliance rates, specific sites/settings involved in the study and other contextual issues | ✔ | 22 |
| Overall Evidence | 22 | General interpretation of results in the context of current evidence and current theory | ✔ | 29 |
