# Supplementary material for: Initial Findings from a Novel School-Based Program, EMPATHY, Which May Help Reduce Depression and Suicidality in Youth
Source: PLoS One. 2015 May 14;10(5):e0125527. doi: 10.1371/journal.pone.0125527 (PMC4431804; doi:10.1371/journal.pone.0125527)
Supplement: S1 Protocol — This shows the original protocol approved by the ethics review committee in September 2013, as well as the subsequent changes made which were approved in January 2014. These changes relate in large degree to a change in the measurement tools, necessitated by a desire to utilize screening tools, and cognitive behavioural treatment (CBT) tools, that would fit within the paradigms established in the study, i.e. being free, brief, and available to use with the technology available to the study and the schools. It can be seen that there varied considerably from the initial tools that were approved for use. (PDF) [file pone.0125527.s002.pdf]

***COMPREHENSIVE PROGRAM FOR YOUTH MENTAL HEALTH***


**Principal Investigator:** *Peter Silverstone*
*Professor Department of Psychiatry, University of Alberta*
*Scientific Director, Strategic Clinical Network on Addiction and Mental Health, Alberta Health Services*

**Co-Investigator:** *Katherine Rittenbach*
*Assistant Scientific Director, Strategic Clinical Network on Addiction and Mental Health, Alberta Health Services*


**Sponsor:** *Addiction and Mental Health Strategic Clinical Network*
*Alberta Health Services*
*10405 Jasper Avenue*
*Edmonton, AB T5J 3N4*

**Protocol number:** AMH–SCN–02

## Table of Contents

**STUDY SUMMARY** ... **3**

***1 INTRODUCTION*** ... **4**

1.1 BACKGROUND ... 4

***2 STUDY DESIGN*** ... **6**

2.1 PRIMARY STUDY OBJECTIVES ... 7

**3 STUDY DESIGN** ... **7**

3.1 GENERAL DESIGN ... 7
3.2 SITE LOCATION ... 7
3.3 SCREENING ... 8
3.4 RESILIENCY PROGRAM ... 8
3.5 ONLINE INTERVENTIONS ... 8
3.6 FURTHER STAGES ... 8
3.7 DATA SECURITY, DATA COLLECTION, AND COST ANALYSIS ... 8
3.8 RANDOMIZATION AND ANALYSIS PLAN ... 9
3.9 ASSIGNING SUBJECTS TO TREATMENT GROUPS ... 9
3.10 SUICIDALITY ... 9
3.11 STUDY WITHDRAWAL AND COMPLETION RATES ... 10

**4 STATISTICAL PLAN** ... **10**

4.1 SAMPLE SIZE DETERMINATION ... 10

**5 DATA HANDLING AND RECORD KEEPING** ... **10**

5.1 CONFIDENTIALITY ... 10

**6 ETHICAL CONSIDERATIONS** ... **11**

**7 REFERENCES** ... **12**

**Study Summary**

| | |
|---|---|
| Title | Comprehensive Program for Youth Mental Health Proposal |
| Methodology | Controlled |
| Study Duration | School Year (9 months); run for 3 consecutive years |
| Study Center(s) | Multi-centre (2 high schools and 3 associated feeder middle/junior high schools) |
| Objectives | • Decreased rates of depression and suicide (as well as rates of suicide attempts)<br>• Decreased use of alcohol, tobacco, and other drugs, with additional downstream benefits such as reduced rates of fetal alcohol spectrum disorder (FASD) and cancer<br>• Decreased school drop-out rates<br>• Decreased rates of interactions with the justice system<br>• Decreased costs across a range of ministries (health, education, justice, human services), both for youth as well as their families who have been involved in this program |
| Number of Subjects | The total number of participants on who data will be collected will be determined by school enrolment. Inclusion in the data collection for the top 10% will be determined by parental consent and participant assent |
| Inclusion Criteria | The intervention group will be the top 10% of all students participating in the school screening. |
| Exclusion Criteria | Lack of consent, lack of assent, lack of the ability to read and understand the screening tools or intervention tools |
| Study Interventions | 1). Resiliency program – Op Volle Kracht (OVK)<br>2). Screening Module:<br>The screening tool has been created to fit during 1 class period (maximum of 50 questions) and includes the questions from:<br>Depression screening – PHQ-A<br>Anxiety screening – Hospital Anxiety and Depression Scale Score<br>Substance misuse screening – CRAFFT Substance Abuse Screening Test<br>Quality of life screening – KidScreen 10<br>Self Esteem – Rosenberg scale<br>3). In School Treatment Module:<br>Depression – Smart, Positive, Active, Realistic X-factor thoughts (SPARX) and/or MoodGYM<br>Anxiety – BRAVE online and Camp-Cope-A-Lot<br>Substance misuse – Leave the Pack Behind, AlbertaQuits services and Climate Schools: Alcohol<br>Body Image – Student Bodies<br>4). A Youth Depression Treatment Pathway developed and tested in Alberta.<br>5). Referral to Primary Care or Specialty Care |

## 1 Introduction

This document is a protocol for a human research study. This study is to be conducted according to Canadian and international standards of Good Clinical Practice (International Conference on Harmonization guidelines), the Declaration of Helsinki (2008 amendment, Seoul, Korea), applicable government regulations and Institutional research policies and procedures.

The majority of addiction and mental health problems seen in adults present first in youth, and there is strong evidence that prevention and early identification approaches in childhood and youth can mitigate some of these risks. The Mental Health Commission released a detailed report in May 2012 which reviewed all of the evidence to date. They stated that "*reducing the impact of mental health problems and illnesses and improving the mental health of the population require promotion and prevention efforts in everyday settings where the potential impact is greatest*", specifically describing that this should occur in schools. Supporting this suggestion is strong evidence that appropriate school interventions can have a dramatic impact on reducing the frequency of youth addictions and mental health problems. Depression, which underlies most suicides, is also amenable to interventions using specific interventions.
This program is outcome-driven and designed to address both prevention of mental health and addiction problems as well as early identification of them.

### 1.1 Background

*The Burden of Depression*
There is evidence that prevention at several different stages in childhood can mitigate the risk of mental illness and addiction, this project is focused on prevention, early identification and rapid treatment in the middle / junior high school period.

According to 2006/07 analysis of all diagnostic groups in Alberta, depression treatment exhibited the following characteristics:

- **High variation:** the cost of treatment between cases is highly variable
- **High cost:** the overall cost of treatment is significant
- **High volume:** the number of patients per year is significant

Due to the overall burden of depression, and the high variability of treatment that is offered, the Addiction & Mental Health Strategic Clinical Network is developing a comprehensive prevention and treatment program for youth that can be spread across Alberta. Establishing evidence based best practices for the prevention and treatment of youth mental illnesses can potentially have major impacts, both for individuals and for the community.

*Alcohol and other drug use*
It is well recognized that Alcohol and other drugs use are significant health burdens in their own right. In addition, addictions can begin as early as adolescence. In the present study we aim to achieve several goals related to this use, but primarily this is to help the program staff identify the use of both of these drugs by the students, and then provide information and treatment options.

All students will complete the CRAFFT Substance Abuse Screening Test which covers both alcohol and other drug abuse.

The present study will be able to evaluate the results of school interventions and can be adjusted to best meet the needs of diverse communities across Alberta.

*Resiliency*

It has long been recognized that there is variation in long term outcomes for those at high risk of mental health illnesses. Research into the cause of these variations focused on resiliency as a concept and research subject (Masten & Tellegen, 2012).One of the most widely used, and best studied, prevention programs for depression is the Penn Resiliency Program (PRP), which was developed in 1990 by Dr. Jane Gillham (Cardemil et al., 2007; Cutuli et al., 2006 and Gillham & Rievich, 1999). Findings from those studies have been generally positive and an updated version has been developed in Holland over a 4-year period by Dr. Rutger Engels, in close collaboration with Dr. Gillham. This updated program is called “Op Volle Kracht” (OVK) which translates to On Full Power and has demonstrated very positive results (Tak et al., 2012). This program contains a cognitive behavioural therapy (CBT) component designed to modify negative thoughts and feelings as well as a social problem-solving component.

*Evidence for Internet-based interventions*

One of the major treatments for mild-moderate depressive disorders is cognitive behavioural therapy (CBT). It has been possible to operationalize some of this treatment and internet-based approaches to this have been developed. The most widely examined in adolescents was developed in New Zealand and is termed “Smart, Positive, Active, Realistic, X-factor thoughts (SPARX)” (https://sparxs.org.nz) (Merry et al., 2012).

Studies comparing internet based interventions to wait lists for treatment of anxiety in youth demonstrate improved patient satisfaction and decreases in anxiety symptoms (Spence et al., 2006). Specific studies on the tool proposed in this protocol, Camp Cope-a-lot, is a computer assisted program that shows sustained decreases in anxiety levels in youth (Khanna & Kendall, in press).

One treatment for alcohol abuse in school aged people is the Climate Schools Course. This is an evidence based online course developed in Australia (Newton et al., 2012).

*Evidence for Clinical Pathways*

There has been a growing awareness in health care circles that certain high frequency, high cost addiction and mental health disorders may be best addressed via the systemic adoption of clinical pathways. A clinical pathway is defined as “a multidisciplinary outline of anticipated care, placed in an appropriate timeframe, to help a patient with a specific condition or set of symptoms move progressively through a clinical experience to positive outcomes.” (Middleton, Barnet & Reeves, 2001). A clinical pathway incorporates guidelines, protocols and evidence informed best practice into everyday use for the patient and family.

A detailed Adolescent Depression Pathway was developed in Alberta. Pilot testing of this pathway in a specialized clinic has shown good acceptance rates, although there were no control groups. The present study has been designed to incorporate this intervention and monitor responses before wider adoption of this Adolescent Depression Pathway.

## 2 *Study Design*

This study is designed to measure objective results at the High School level, the study is designed with internal controls as the students who participate in this protocol will enter the high schools over time (see figure 1).

| | | | |
|---|---|---|---|
| **Year #1** | | | |
| | | | Baseline |
| High School | Age | Grade | |
| | 17 | 12 | Screening |
| | 16 | 11 | Screening |
| | 15 | 10 | Screening |
| Junior High School | Age | Grade | |
| | 14 | 9 | Resiliency and Screening |
| | 13 | 8 | Resiliency and Screening |
| | 12 | 7 | Resiliency and Screening |
| **Year #2** | | | |
| High School | Age | Grade | |
| | 17 | 12 | Screening |
| | 16 | 11 | Screening |
| | 15 | 10 | Screening (+ Resiliency x1) |
| Junior High School | Age | Grade | |
| | 14 | 9 | Resiliency and Screening |
| | 13 | 8 | Resiliency and Screening |
| | 12 | 7 | Resiliency and Screening |
| **Year #3** | | | |
| High School | Age | Grade | |
| | 17 | 12 | Screening |
| | 16 | 11 | Screening (+ Resiliency x1) |
| | 15 | 10 | Screening (+ Resiliency x2) |
| Junior High School | Age | Grade | |
| | 14 | 9 | Resiliency and Screening |
| | 13 | 8 | Resiliency and Screening |
| | 12 | 7 | Resiliency and Screening |
| **Year #4** | | | |
| High School | Age | Grade | |
| | 17 | 12 | Screening (+ Resiliency x1) |
| | 16 | 11 | Screening (+ Resiliency x2) |
| | 15 | 10 | Screening (+ Resiliency x3) |
| Junior High School | Age | Grade | |
| | 14 | 9 | Resiliency and Screening |
| | 13 | 8 | Resiliency and Screening |
| | 12 | 7 | Resiliency and Screening |

There are other control groups that will be considered, including any students that enter the high schools that have not received the resiliency training in the middle schools (either because they are new to the district or attended a school that did not offer the resiliency training).

Further comparisons will be made between the students identified as “high risk” who take part in the in school interventions and those who do not (due to lack of consent).

### *2.1 Primary Study Objectives*

The primary study objectives for this study are:

1. **Decreased rates of depression and suicide (as well as rates of suicide attempts)**
2. Decreased use of alcohol, tobacco, and other drugs, with additional downstream benefits such as reduced rates of fetal alcohol spectrum disorder (FASD) and cancer
3. Decreased school drop-out rates
4. Decreased rates of interactions with the justice system
5. Decreased costs across a range of ministries (health, education, justice, human services), both for youth as well as their families who have been involved in this program

It is expected that the program will decrease costs across a range of ministries (health, education, justice, human services), both for youth as well as their families who have been involved in this program.

## 3 Study Design

### *3.1 General Design*

This is a controlled study in youth 11 to 17 years old attending a participating school, who are cognitively capable of independently understanding and confirming their desire to take part and whose parents/guardians also provide informed consent for participation. Sub-analyses may be completed to determine if sex, socioeconomic background or race influence the effectiveness of the interventions. Currently one school district is involved in the study and 2 High Schools and 3 Middle Schools will take part. However, this study could be expanded to other school districts.

**Figure 1. Timing of study**

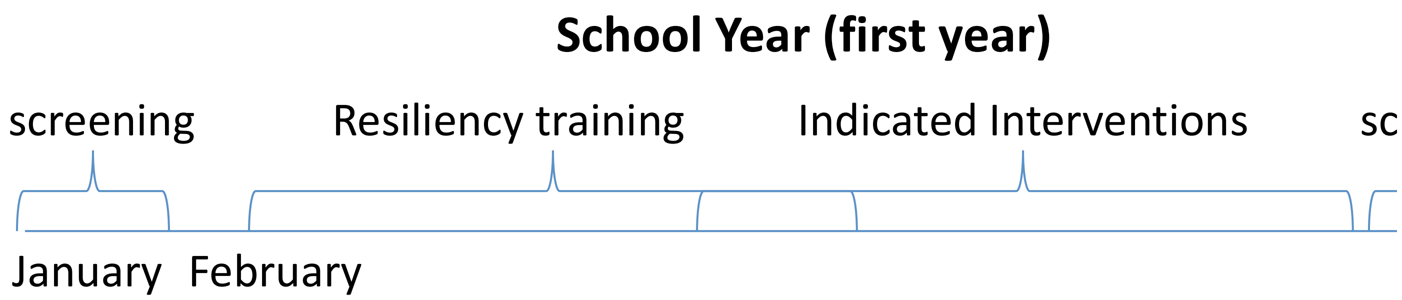


### *3.2 Site location*

It is intended that 3 High Schools and 2 Middle Schools in Red Deer Public School District will participate in this study. The leadership team of the school district have been part of the design of this study and are actively engaged.

### 3.3 Screening

Screening will be completed within the classroom, during one class period all students in the class will complete the screening tools electronically. The screening tool has been created to be completed in one class period (maximum of 50 questions) and consists of 5 domains (see attachment 1). The 5 domains will be randomized during screening (i.e. each student will take them in random order) in order to minimize survey fatigue effects.:

Depression screening – PHQ-A
Anxiety screening – Hospital Anxiety and Depression Scale
Substance misuse screening – CRAFFT Substance Abuse Screening Test
Quality of life screening – KidScreen 10
Self Esteem – Rosenberg scale

Based on the results of the screening (see attachment 2), the school will send the information letter and consent form to the parents of high risk students in the Junior High and High Schools for the principal investigator (PI). The parents will be able to contact the researchers with any questions regarding the study. Any students that demonstrate significant risk on the self-harm measures will be referred to either primary care or specialty care immediately depending upon severity.

### 3.4 Resiliency Program

The Op Volle Kracht (OVK) program is a dutch translation and update of the well established Penn Resiliency Program (PRP). This program has been studied in multiple randomized clinical trials and is a group intervention that teaches cognitive-behavioral and social problem-solving skills. OVK uses lessons that fit well in most school schedules that focus on increasing resiliency against depression and specifically has been designed to modify negative thoughts and feelings. The program will be adjusted for each grade level by a team of experts in order to ensure that it is appropriate for each grade it is presented to. The program will be led by a study staff member trained in the program.

### 3.5 Online Interventions

Those students who screening identifies as "high risk" will be invited to participate in guided internet interventions led by study staff. These interventions include:

- Depression – Smart, Positive, Active, Realistic X-factor thoughts (SPARX)
- Anxiety – BRAVE online and Camp-Cope-A-Lot
- Substance misuse – Leave the Pack Behind, AlbertaQuits services and Climate Schools: Alcohol
- Body Image – Student Bodies

### 3.6 Further Stages

The study staff will be embedded in the schools and will be able to help navigate the health care system for the student and family when necessary. This may include connections to the local Primary Care Network / Family Care Centre and to specialty care.

### 3.7 Data Security, Data Collection, and Cost analysis

Collection of all data will be compliant with the Health Information Act of Alberta. Electronic data will be transmitted in an encrypted manner over the internet. As soon as student data is transmitted, neither the student nor the study staff will have further access to that (or any other)

information from the screening device. It will not be possible for any student to view their information, or anyone else's, as soon as they have completed each form.

The students will be asked for their name and grade during screening in order to collect the identifiers for the study (the Alberta Health Care Number and student ID), but no other identifiers will be collected in the database.

All collected information will be transferred immediately to secure servers and kept encrypted until collected by AHS staff. This will be independently validated on a frequent basis. All data will be stored by either Red Deer Public Schools or AHS.

On an anonymized basis the individual Alberta health care numbers will be linked to other databases to allow determination of the primary objectives for each individual. All linkages will be governed by appropriate regulations and confidentiality will be maintained. The linkages may include with health information regarding Emergency Room visits, justice department information, and the education system.

### *3.8 Randomization and Analysis plan*

There is no randomization in this study.

For statistical analysis, the change in PHQ-A in the high schools between groups who have had resiliency training and groups who haven't is the primary outcome variable. The main analysis will utilize linear regression, with indicator variables identifying the groups. We will evaluate group by school interactions using indicator variables for school and cross-product interaction terms to confirm that group effects are consistent across the schools. Other analyses of interest will include the comparison of screening results before and after resiliency training and internet interventions in the Middle School students and comparison of screening results between students in the top 10% who participate in the interventions and those who do not. The other analyses will be secondary, as will subgroup analyses, e.g. by age, sex etc.

### *3.9 Assigning Subjects to Treatment Groups*

All Junior High/Middle school students will receive resiliency training and screening unless absent for the scheduled days. Those whose screening suggests existing problem or individual at high-risk, as defined by being in the top 10% of scores, will be offered the school-based intervention unless they demonstrate significant risk on the self-harm measures in which case they will be referred primary care or specialty care depending upon severity.

Timing for recruitment
A dedicated study team will be involved at the schools and will be responsible for screening Junior High/Middle School and High School's students and coordinating the organization of school based interventions for those students that are identified as high risk.

### *3.10 Suicidality*

If any subject expresses significant suicidal thoughts or feelings, the patients parents will be immediately notified and appropriate action taken.

### *3.11 Study Withdrawal and completion rates*

Any participant can withdraw at any time for any reason.

We anticipate that study completion rates will be 70%. However, it should be noted that follow up information will be collected for all participants, even if they do not complete, in part to assess the effect of a universal resiliency program on the entire student body.

## 4 Statistical Plan

### *4.1 Sample Size Determination*

The sample size is presumed to be the population of the Red Deer High Schools which is approximately 3,000 individuals. A power analysis suggests that with a 95% confidence level a difference of 4% in the rate of depression between years can be detected ($p<0.05$). As this study is designed to produce significant reductions in the rate of depression and other mental health issues in the High Schools, the ability to determine differences as small as 4% is considered sufficiently powered.

Other analyses that are planned include the comparison of the screening tool results for the Junior High School students before and after the resiliency program and in school interventions. This will be done by comparing the average of the first 2 screening results to the screening results from June. In a previous study there was a decrease of only 2 points in the PHQ-9 at 6 weeks and 6 months when no specific treatment was given (Christensen et al., 2011). Previous studies in adults have shown a 10 point decrease due to treatment with the adult depression pathway. Although there have not been studies utilizing the PHQ-9 with the internet treatment in adolescents, previous studies have found that in adults this produces results that are somewhat similar to other treatments. Therefore, we conservatively estimate that the differences in PHQ-A scores after the internet intervention will be 6 points (this is 75% of the observed difference in adults).

A power analysis suggests that with a 95% confidence level the sample size needed is 93. The anticipated number of students who score in the top 10% on the screening tool will be 200 individuals (10% of the estimated student population of 2000 in Red Deer middle schools). Since we conservatively anticipate that 70% of students will have complete consents and will complete the intervention, this will give an analysis of 140 students per group. Thus, this study is sufficiently powered to show a decrease in depression rates after guided internet interventions in schools.

## 5 Data Handling and Record Keeping

### *5.1 Confidentiality*

Information about study subjects will be kept confidential and managed according to the requirements of the Health Information Act of Alberta. Those regulations require a signed parental consent informing the subject of the following:

- What protected health information will be collected from subjects in this study
- Who will have access to that information and why
- Who will use or disclose that information
- The rights of a research subject to revoke their authorization for use of their protected health information.

## 6 Ethical Considerations

This study is to be conducted according to International standards of Good Clinical Practice (International Conference on Harmonization guidelines), the Declaration of Helsinki (2008 amendment, Seoul, Korea), applicable government regulations and Institutional research policies and procedures.

This protocol and any amendments will be submitted to a properly constituted independent Ethics Committee (EC) in agreement with local legal prescriptions, for formal approval of the study conduct. The decision of the EC concerning the conduct of the study will be made in writing to the investigator before commencement of this study.

All participants for this study will be provided a parental/guardian consent form describing this study and providing sufficient information for participants and their parent/guardians to make an informed decision about their participation in this study. This consent form will be submitted with the protocol for review and approval by the EC/IRB for the study. The formal consent of a subject, using the EC-approved consent form, must be obtained before that subject undergoes any study procedure, and the consent form must be completed by the subject.

## 7 References

Cardemil, E.V., Reivich, K.J., Beevers, C.G., Seligman, M.E.P. & James, J; 2007; *The prevention of depressive symptoms in low-income, minority children: Two-year follow-up;* Behaviour Research and Therapy; 45: 313-27

Christensen, H., Aiken, A., Batterham, P.J., Walker, J., Mackinnon, A.J., French, M. & Hickie, I.B.; 2011; *No clear potentiation of antidepressant medication effects by folic acid + vitamin B12 in a large community sample;* Journal of Affective Disorders; 130: 37-45

Cutuli, J.J., Chaplin, T.M, Gillham, J.E., Reivich, K.J. & Seligman, M.E.P.; 2006; *Preventing Co-Occuring Depression Symptoms in Adolescents with Conduct Problems: The Penn Resiliency Program;* Annuals of New York Academy of Science; 1094: 282-6

Gillham, J.E. & Reivich, K.J.; 1999; *Prevention of depressive symptoms in schoolchildren: A research update;* Psychological Science; 10: 461-2

Khanna, M.S. & Kendall, P.C.; in press; *Computer-assisted cognitive-behavioral therapy for child anxiety: results of a randomized clinical trial;* Journal of Consulting and Clinical Psychology;

Newton, N.C., Teeesson, M., Barrett, E.L., Slade, T., & Conrad, P.J.; 2012; *The CAP study, evaluation of integrated universal and selective prevention strategies for youth alcohol misuse: study protocol for a cluster randomized controlled trial;* BMC Psychiatry; 12: 118

Masten, A.S. & Tellegen, A.; 2012; *Resilience in development psychopathology: Contributions of the Project Competence Longitudinal Study;* Development and Psychophathology; 24: 345-61

Merry, S.N., Stasiak, K., Shepherd, M., Frampton, C., Fleming, T., & Lucassen, M.F.; 2012; *The effectiveness of SPARX, a computerized self help intervention for adolescents seeking help for depression: randomized controlled non-inferiority trial;* British Medical Journal; 344: e2598

Spence, S.H., Holmes, J.M., March, S. & Lipp, O.V.; 2006; *The feasibility and outcome of clinical plus internet delivery of cognitive-behaviour therapy for childhood anxiety;* Journal of Consulting and Clinical Psychology; 74: 614-21

Tak, Y.R., Van Zundert, R.MP, Kuijpers, R. CWM, Van Vlokhoven, R.S., Rensink, H.FW & Engels, R. CME; 2012; *A randomized controlled trial testing the effectiveness of a universal school-based depression prevention program 'Op Volle Kracht' in the Netherlands,* BMC Public Health; 12: 21

Attachment A: Screening Tool Questions

## Attachment B: Screening Tool Scoring Description

EMPATHY screening scoring framework

There are 5 domains in the screening questions.

1. Quality of Life (11 questions)
2. Drugs/Alcohol/Tobacco (minimum 6 questions, maximum 11 questions)
3. Depression (11 questions scored, 13 total)
4. Self Esteem (10 questions)
5. Anxiety (7 questions)

To normalize the data for the calculation of the top 10% we propose that each domain is given equal weight (20% of the score):

Quality of life will be scored 1-5 and summed for a maximum score of 55 = QoL
Drugs/Alcohol/Tobacco will be scored as either 0 for no or 1 for yes for a maximum score of 11 = DAT
Depression will be scored from 0-3 for 11 of the questions, 0-1 for one question for a maximum score of 34 (the other two questions are around suicide and will be automatic signalers for intervention)= Dep
Self Esteem will be scored from 0 - 3 for a maximum score of 30 = SE
Anxiety will be scored from 0-3 for a maximum score of 21 = Anx

**Screening score = {20 – [(QoL - 11) x 20/44]} + (DAT x 20/11) + (Dep x 20/34) + [20 – (SE x 20/30)] + (Anx x 20/21)**

**Range will be: (0+0+0+0+0) = 0 to (20+20+20+20+20) = 100**

Example: child with QoL: 44; DAT: 3; Dep: 8; SE: 25; Anx: 18 (drinks, high QoL and SE, no Dep, some Anx)
score = {20-[(44-11) x 20/44]} + (3 x 20/11) + (8 x 20/34) + [20 – (25 x 20/30)] + (18 x 20/21)
score = 5 + 4.45 + 4.70 + 3.33 + 17.14 = 34.62

Example 2: child with QoL: 20; DAT: 0; Dep: 14; SE: 15; Anx: 14 (no drinking/drugs, low QoL and SE, moderate Dep and slightly anxious)
Score = {20-[(44-20) x 20/44]} + (0 x 20/11) + (14 x 20/34) + [20 – (15 x 20/30)] + (14 x 20/21)
Score = 9.1 + 0 + 8.24 + 10 + 13.33 = 40.67

Example 3: child with QoL: 10; DAT: 6; Dep: 20; SE: 9; Anx: 24 (drinks, smokes marijuana and tobacco, low QoL and SE, Depressed and anxious)
Score = {20-[(44-10) x 20/44]} + (6 x 20/11) + (20 x 20/34) + [20 – (9 x 20/30)] + (24 x 20/21)
Score = 4.55 + 10.9 + 11.76 + 14 + 22.85 = 64.06
